# Genome-Wide Identification and Validation of Reference Genes in Infected Tomato Leaves for Quantitative RT-PCR Analyses

**DOI:** 10.1371/journal.pone.0136499

**Published:** 2015-08-27

**Authors:** Oliver A. Müller, Jan Grau, Sabine Thieme, Heike Prochaska, Norman Adlung, Anika Sorgatz, Ulla Bonas

**Affiliations:** 1 Institute for Biology, Department of Genetics, Martin Luther University Halle-Wittenberg, Halle (Saale), Germany; 2 Institute for Informatics, Department of Bioinformatics, Martin Luther University Halle-Wittenberg, Halle (Saale), Germany; Shanghai Jiao Tong University, CHINA

## Abstract

The Gram-negative bacterium *Xanthomonas campestris* pv. *vesicatoria* (*Xcv*) causes bacterial spot disease of pepper and tomato by direct translocation of type III effector proteins into the plant cell cytosol. Once in the plant cell the effectors interfere with host cell processes and manipulate the plant transcriptome. Quantitative RT-PCR (qRT-PCR) is usually the method of choice to analyze transcriptional changes of selected plant genes. Reliable results depend, however, on measuring stably expressed reference genes that serve as internal normalization controls. We identified the most stably expressed tomato genes based on microarray analyses of *Xcv*-infected tomato leaves and evaluated the reliability of 11 genes for qRT-PCR studies in comparison to four traditionally employed reference genes. Three different statistical algorithms, geNorm, NormFinder and BestKeeper, concordantly determined the superiority of the newly identified reference genes. The most suitable reference genes encode proteins with homology to PHD finger family proteins and the U6 snRNA-associated protein LSm7. In addition, we identified pepper orthologs and validated several genes as reliable normalization controls for qRT-PCR analysis of *Xcv*-infected pepper plants. The newly identified reference genes will be beneficial for future qRT-PCR studies of the *Xcv*-tomato and *Xcv*-pepper pathosystems, as well as for the identification of suitable normalization controls for qRT-PCR studies of other plant-pathogen interactions, especially, if related plant species are used in combination with bacterial pathogens.

## Introduction

The analysis of gene transcription profiles is a powerful tool to uncover the roles of specific genes in cellular processes and to place them into regulatory networks. Quantitative reverse transcription PCR (qRT-PCR), also termed real-time RT-PCR, is the method of choice to analyze changes in gene transcription because of its high sensitivity, large dynamic range and accuracy [1]. The reliability of results strongly depends on suitable reference genes for normalization which should be stably expressed under the experimental conditions used. Housekeeping genes encoding, e.g., actin, glyceraldehyde-3-phosphate dehydrogenase (GAPDH) and ribosomal RNAs, are generally assumed to represent suitable normalization controls [2]. However, a number of studies reported that transcription of housekeeping genes can fluctuate considerably under certain experimental conditions, even if expression is constant in other cases ([3] and references therein). This illustrates the necessity to systematically validate reference genes for specific experimental conditions to avoid misinterpretation of qRT-PCR results [3, 4].

The interaction of plants with pathogens induces dramatic changes in plant transcription patterns. In most cases, the plant withstands pathogen attacks by inducing innate immune responses, associated with transcriptional reprogramming, e.g., the induction of pathogenesis-related (*PR*) genes [5–7]. Specialized pathogens, however, can suppress plant immunity and successfully colonize the host. Infection is accompanied by transcriptional changes of numerous plant genes including those involved in basal cell processes [7–12]. For example, in maize seeds infected by fungi genes involved in metabolism, energy and protein synthesis are prevalently down-regulated, including classical housekeeping genes like GAPDH [9]. The bacterial pathogen *Pseudomonas syringae* pv. *tomato* represses cell wall and photosynthetic genes in Arabidopsis plants [12]. Similar results were obtained in sweet orange and peach infected with *Xanthomonas citri* supsp. *citri* and *X*. *arboricola* pv. *pruni*, respectively [8, 11].

Recently, there were a number of reports validating reference genes in different plant species after infection with fungi, oomycetes, viruses or bacteria [13–31], or suffering from plant and animal parasites [32–36]. Among the genes most often found to be suitable normalization controls under biotic stress conditions were genes encoding actin [13, 23, 24, 30, 34, 35], glyceraldehyde 3-phosphate dehydrogenase (GAPDH) [15, 16, 27, 28, 30], β-tubulin [17, 25, 28, 32] and elongation factor 1α (EF-1α) [21, 34–36]. However, a major drawback of most studies is the selection of reference gene candidates based on “the usual suspects”, i.e., genes with known or suspected housekeeping roles. Such a biased approach might miss the optimal internal control. This idea is supported by whole-transcriptome analyses in different plant species and different experimental setups that, together with qRT-PCR studies, identified genes differing from the traditional housekeeping genes as most stably transcribed [37–43].

Our lab studies the interaction of the phytopathogenic γ-proteobacterium *X*. *campestris* pv. *vesicatoria* (*Xcv*) with its solanaceous hosts, tomato (*Solanum lycopersicum*) and pepper (*Capsicum annuum*). *Xcv* causes bacterial spot disease which results in defoliation and severely spotted fruits, both of which lead to massive yield losses, especially in regions with a warm and humid climate [44]. An essential pathogenicity factor of *Xcv* is the type III secretion (T3S) system that translocates bacterial effector proteins into the plant cell cytosol. Although the molecular function of many *Xcv* type III effectors is unknown, several suppress host defenses elicited upon recognition of pathogen-associated molecular patterns (PAMPs), i.e., PAMP-triggered immunity (PTI) [45]. A well-characterized effector family from *Xanthomonas* are TAL (transcription activator-like) effectors [46]. The type member AvrBs3 from *Xcv* binds to plant gene promoters and activates the transcription of *UPA* (upregulated by AvrBs3) genes in pepper and other solanaceous plants resulting in hypertrophy, i.e., enlargement, of mesophyll cells [47, 48]. In resistant pepper plants, *UPA* genes include the *Bs3* resistance gene leading to the specific elicitation of the hypersensitive response (HR), a rapid, localized programmed cell death at the infection site, that is a hallmark of effector-triggered immunity (ETI) [49].

Since we are interested in transcriptome changes during pathogen attack, we first analyzed the results of two genome-wide microarray screens of tomato cv. MoneyMaker (MM) to identify reference gene candidates suitable for qRT-PCR analysis of *Xcv*-infected (pathogenic and non-pathogenic strains) compared to unchallenged plants. Validation by qRT-PCR revealed 11 novel tomato reference genes. In addition, we identified the pepper orthologs of these genes and found several to be suitable normalization controls for qRT-PCR analyses in pepper during biotic stress.

## Material and Methods

### Plant material and inoculations

Tomato (*Solanum lycopersicum*) plants of cultivar (cv.) MoneyMaker and pepper (*Capsicum annuum*) cv. ECW-30R plants were grown in the greenhouse under standard conditions (day and night temperatures of 23°C and 19°C, respectively, for tomato, and 25°C and 19°C for pepper, with 16 h light and 40 to 60% humidity). For qRT-PCR studies, tomato and pepper plants were transferred to a Percival growth chamber (Percival Scientific, Perry, USA) three days before inoculation. Mature leaves of seven-week-old tomato and pepper plants were inoculated with mock (10 mM MgCl_2_) or *Xcv* (5×10^8^ cfu/ml in 10 mM MgCl_2_) using a needleless syringe.

### Bacterial strains and growth conditions

*Xcv* strains 85–10 [50] and 85–10Δ*hrcN* [51] were grown at 30°C on NYG (nutrient yeast glycerol) agar plates [52] supplemented with appropriate antibiotics. Plasmids pLAT211 (*avrBs4* in pLAFR6 [53]) and pGGX1:avrBs3 [54] were introduced into *Xcv* by conjugation, using pRK2013 as helper plasmid in triparental matings [55].

### Microarray analyses

For microarray studies, 12 tomato plants were inoculated per experiment. To minimize differences in gene expression due to leaf-to-leaf variability, *Xcv* strains and 10 mM MgCl_2_, respectively, were infiltrated into the same leaves. Four leaf discs (0.5 cm diameter) per inoculum and leaf were harvested, immediately frozen in liquid nitrogen and stored at -80°C. In the first study, *Xcv* 85–10 and 85–10Δ*hrcN* were inoculated; leaf material was harvested 45 min and 6, 10 and 24 hours post infiltration (hpi). Leaf material of four plants was pooled for each time-point (16 leaf discs per sample, three technical replicates). In the second study, 85–10Δ*hrcN* and 10 mM MgCl_2_ were infiltrated and leaf material was harvested at 0, 4, 8 and 16 hpi and pooled as above. In addition, four leaf discs per plant were harvested as control before treatment. This was performed three times independently with four plants each (biological replicates). The experimental setup is summarized in S1 Fig.

Total RNA was extracted using the QIAGEN RNeasy Plant Mini Kit (QIAGEN, Hilden, Germany) and treated with DNase I (Roche, Mannheim, Germany) for 30 min. Approximately 1.5 μg total RNA was sent to Source BioScience (Berlin, Germany) for cDNA synthesis and microarray hybridizations.

For the tomato whole-genome chip (Source BioScience), oligonucleotides for 34,383 annotated tomato genes [according to the international tomato annotation group (ITAG, version 2.3)] were spotted on Agilent custom arrays. Five 50-bp oligonucleotides per gene were tested on an Agilent custom array 4x180K, and a set of suitable oligos was chosen for the final chip. Due to space limitations (8x60K), 25,985 randomly chosen genes were represented twice with different oligonucleotides, whereas 8398 genes were represented by one oligonucleotide each. Finally, seven identical 8x60K chips were used for sample analysis. Different chips were hybridized with biological and technical replicates, respectively. cDNA synthesis, labelling, hybridization, washing, scanning and data collection was performed by Source BioScience according to Agilent standard protocols.

### Data processing and statistical analyses

Microarray raw data (column "gProcessedSignal") were analyzed by the statistical software R [56]. All experiments of one study (treatments, time points and replicates) were normalized by quantile normalization on the probe level using the "preprocessCore" R package (version 1.26.1, http://www.bioconductor.org/packages/release/bioc/html/preprocessCore.html). For each gene, values for transcript accumulation were obtained as the arithmetic mean of the intensities of all probes representing the gene. The coefficient of variation (CV) was computed for each gene as the standard deviation of its transcript levels across all experiments divided by its mean transcript level. To evaluate the similarity of expression patterns in biological and technical replicates, normalized log-expression values of the individual experiments were clustered hierarchically using the R function hclust [56]. The distance between the expression vectors of experiments was determined as one minus the Pearson correlation of log-expression values using the R function cor.dist from the bioDist package of the Bioconductor suite [57]. Clustering was performed using complete linkage, which yields compact clusters with high intra-cluster correlations. Dendrograms were plotted using the specific plot function of the R class hclust [56].

### Quantitative reverse transcription polymerase chain reaction (qRT-PCR)

Templates for qRT-PCR were produced as follows: three to four leaf discs (1.3 cm diameter) from different plants infiltrated with *Xcv* and MgCl_2_, respectively, were pooled for RNA isolation using the QIAGEN RNeasy Plant Mini Kit. Oligo-dT- and random hexamer-primed cDNA was synthesized with the Maxima First Strand cDNA Synthesis Kit (Thermo Scientific, Schwerte, Germany). qRT-PCR was performed on a CFX96 thermal cycler (Bio-Rad, Munich, Germany) using a SYBR Green-based PCR reaction mixture (Absolute Blue qPCR SYBR Green Fluorescein Mix; Thermo Scientific) and 8 ng template cDNA. Oligonucleotide sequences are listed in S1 Table. To compare Ct (cycle threshold) values measured on different plates using different reaction mixtures, automatically calculated thresholds of all plates were set manually to the highest threshold obtained. The efficiency of PCR reactions was determined for each primer pair using a dilution series of template plotted into a standard curve. To ensure amplification specificity, amplicons were subjected to melting curve analysis and analyzed on 1% agarose gels. Transcript levels were determined as technical duplicates of biological triplicates.

### Evaluation of reference gene stability

qRT-PCR data were analyzed using geNorm [58] which is included in the GenEx package (GenEx6 version 3.1.3; http://multid.se), NormFinder [59] and BestKeeper [60].

## Results

### Selection of candidate reference genes for gene expression studies in tomato

To identify reference genes suitable for the analysis of *Xcv*-induced changes in the mRNA levels of tomato genes we evaluated the results of two whole-genome microarray screens. For the first screen, *S*. *lycopersicum* cv. MM plants were inoculated with the *Xcv* wild-type (WT) strain 85–10 and the T3S-deficient derivative 85–10Δ*hrcN*, respectively. Leaf material was harvested 45 min and 6, 10 and 24 hours post infiltration (hpi). In the second screen, *S*. *lycopersicum* cv. MM plants challenged by 85–10Δ*hrcN* inoculation were compared to mock-infiltrated tomato plants, and leaf material was harvested at 0, 4, 8 and 16 hpi. Transcriptional changes of 34,383 annotated tomato genes were analyzed using “Agilent custom arrays“. Hierarchical cluster analysis illustrates similar expression patterns in biological and technical replicates confirming that the experimental treatments worked (S1 Fig). In the first screen, two samples (“85–10; 45 mpi; #2” and “85–10; 6 hpi; #2”) showed aberrant gene expression patterns compared to the corresponding replicates resulting in separate clustering (S1A Fig). Both samples were excluded from further data evaluation.

**Fig 1:**
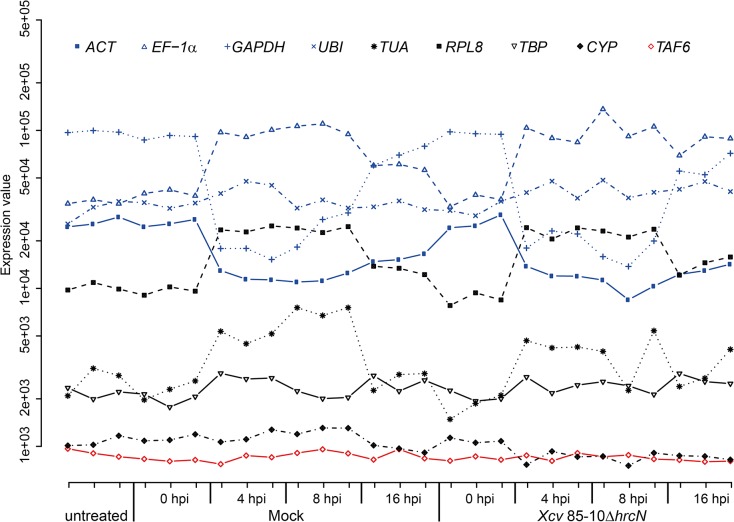
Expression pattern of traditional reference genes in healthy and *Xcv*-infected tomato plants. Leaves of *S*. *lycopersicum* cv. MM plants were untreated or infiltrated with 10 mM MgCl_2_ (mock) and 85–10Δ*hrcN*, respectively. To analyze mRNA accumulation of selected genes leaf material was harvested at 0, 4, 8 and 16 hours post infiltration (hpi). Relative RNA levels of housekeeping genes traditionally employed as references [30, 61–64] were determined by microarray hybridization analysis. Expression values of analyzed plant samples are plotted separately, i.e., three biological replicates per time-point per infiltration. For each gene, expression values were obtained as the mean of the intensities of all probes representing the respective gene. Blue curves represent housekeeping genes that were also analyzed in this study together with novel reference genes. In addition, the most stably expressed gene in the microarray experiment is shown (red curve). *ACT* (*actin*), TC194780a; *EF-1α* (*elongation factor 1α*), SGN-U212845; *GAPDH* (*glyceraldehyde 3-phosphate dehydrogenase*), TC198136a; *UBI* (*ubiquitin*), TC193502a; *TBP* (*TATA binding protein*), SGN-U329249; *RPL8* (*ribosomal protein L8*), X64562; *TUA (α-tubulin*), AC122540; *CYP* (*cyclophilin*), AK326854; *TAF6* (*TFIID subunit 6*), Solyc10g006100.2.1.

The microarray analyses revealed a high variability in the expression patterns of housekeeping genes conventionally used as references in transcript studies [30, 61–64] (Fig 1). To identify the most stably transcribed genes, the coefficient of variation (CV) was determined for each gene, which is defined as the standard deviation of its expression levels across all experiments (treatments and time-points) divided by its mean expression level. Genes with a log_2_ mean expression level below 7 or above 13 were excluded to account for the bigger influence of random noise on low expression values, and for saturation effects of microarrays at high mRNA levels, respectively. Genes with CV values ≤ 0.12 in both microarray studies were ranked by increasing CV in the second screen which delivers more reliable data compared to the first study (biological instead of technical replicates). The best 50 candidate reference genes are listed in Table 1. The tomato sequences were classified based on the functional categories of their *A*. *thaliana* orthologs which were identified by BLASTx [65] against “The Arabidopsis Information Resource” database (TAIR Blast 2.2.8; S2 Fig). Only predicted proteins that displayed minimum 40% amino-acid identity over at least 70% of the tomato sequence were taken into account. This allowed a functional classification of approximately three quarters of the sequences (74%), most of them possessing putative functions in protein expression (transcription and splicing) and turnover (ubiquitination/proteolysis; S2 Fig).

**Table 1:** The 50 most stable tomato genes during *Xcv* infection based on microarray analyses.

Gene ID	CV^a)^	ME	SD	Annotation^b)^
Solyc10g006100.2	0.060	858	52	Transcription initiation factor TFIID subunit 6
Solyc07g062920.2	0.063	623	39	Genomic DNA chromosome 5 TAC clone K19P17
Solyc01g111780.2	0.064	1,254	80	Importin beta-2 subunit
Solyc06g051420.2	0.080	1,537	122	PHD finger family protein
Solyc12g057120.1	0.080	3,234	258	Subunit VIb of cytochrome c oxidase
Solyc01g009290.2	0.082	1,276	105	Polyribonucleotide 5´-hydroxyl-kinase Clp1
Solyc09g018730.2	0.083	2,166	180	Ubiquitin carboxyl-terminal hydrolase family 1 protein
Solyc02g088110.2	0.085	2,367	201	Polypyrimidine tract-binding protein-like
Solyc08g060860.2	0.086	1,032	88	Genomic DNA chromosome 3 P1 clone MSJ11
Solyc09g009640.2	0.087	4,740	414	U6 snRNA-associated Sm-like protein LSm7
Solyc04g015370.2	0.088	2,584	228	Acyl carrier protein
Solyc08g005140.2	0.088	1,192	105	Serine/threonine-protein kinase BUD32
Solyc02g062920.2	0.089	4,256	380	Splicing factor U2AF large subunit
Solyc10g076910.1	0.090	624	56	Pre-mRNA splicing factor ATP-dependent RNA helicase-like protein
Solyc03g121980.2	0.091	1,831	166	Developmentally-regulated GTP-binding protein 2
Solyc01g097140.2	0.092	5,273	486	Dual-specificity tyrosine-phosphatase CDC25
Solyc07g007040.2	0.092	2,611	241	Zinc finger CCCH-type with G patch domain-containing protein
Solyc06g069310.2	0.093	5,571	519	Nuclear transcription factor Y subunit B-6
Solyc03g078020.2	0.094	446	42	Peptide chain release factor 1
Solyc10g078180.1	0.095	1,526	144	Cyclin family protein
Solyc02g089230.2	0.095	2,392	227	DSBA oxidoreductase family protein
Solyc06g036720.2	0.095	1,450	138	HLA-B associated transcript 3 (Fragment)
Solyc01g109620.2	0.095	6,851	652	NADH-quinone oxidoreductase subunit I
Solyc07g064510.2	0.096	6,678	642	Polyadenylate-binding protein 2
Solyc11g071930.1	0.096	639	61	DnaJ homolog subfamily C member 8
Solyc06g084000.2	0.097	1,933	187	Heterogeneous nuclear ribonucleoprotein K
Solyc04g009230.2	0.097	1,799	175	Mitosis protein Dim1
Solyc06g073870.2	0.099	2,349	231	DNA-directed RNA polymerase II subunit RPB4
Solyc09g055760.2	0.099	1,421	141	T-snare
Solyc12g005780.1	0.100	1,138	114	TraB family protein
Solyc04g008610.2	0.101	457	46	Histone acetyltransferase
Solyc04g015300.2	0.101	521	52	Alpha/beta hydrolase
Solyc10g005800.2	0.101	3,475	351	CWC15 homolog
Solyc12g021130.1	0.101	240	24	3-beta-hydroxysteroid dehydrogenase-like
Solyc01g079330.2	0.101	1,160	117	ATP dependent RNA helicase
Solyc07g041550.2	0.101	1,066	108	RNA polymerase-associated protein Ctr9 homolog
Solyc03g059420.2	0.102	1,704	173	Sister chromatid cohesion 2
Solyc11g071950.1	0.102	767	78	Unknown Protein
Solyc12g099570.1	0.103	854	88	Heat shock factor binding protein 2
Solyc10g044900.1	0.103	160	16	CASTOR protein (Fragment)
Solyc10g084270.1	0.103	969	100	Importin α-2 subunit
Solyc06g016750.2	0.103	1,356	140	Transcription factor (Fragment)
Solyc02g092380.2	0.104	699	72	Peptidyl-prolyl cis-trans isomerase
Solyc05g052960.2	0.104	1,149	119	BTB/POZ domain containing protein expressed
Solyc06g009860.1	0.104	1,044	108	Mercaptopyruvate sulfurtransferase-like protein
Solyc10g008950.2	0.104	977	102	Guanylate-binding protein 10
Solyc10g055450.1	0.105	1,503	157	Ubiquitin-protein ligase 4
Solyc05g006580.2	0.105	518	54	Unknown protein
Solyc03g121310.2	0.105	3,802	398	RWD domain-containing protein
Solyc09g010180.2	0.106	1,850	196	TPR repeat-containing protein

^a)^ Coefficient of variation (CV) values for the second microarray study defined as standard deviation (SD) of expression levels of a specific gene across all experiments (treatments, time points, and replicates) divided by its mean expression level (ME). Only genes with a CV value ≤ 0.12 in the first microarray study are listed.

^b)^ Based on the annotation by the international tomato annotation group”(ITAG, version 2.3).

### Evaluation of the expression stability of novel and traditional tomato reference genes

**Fig 2:**
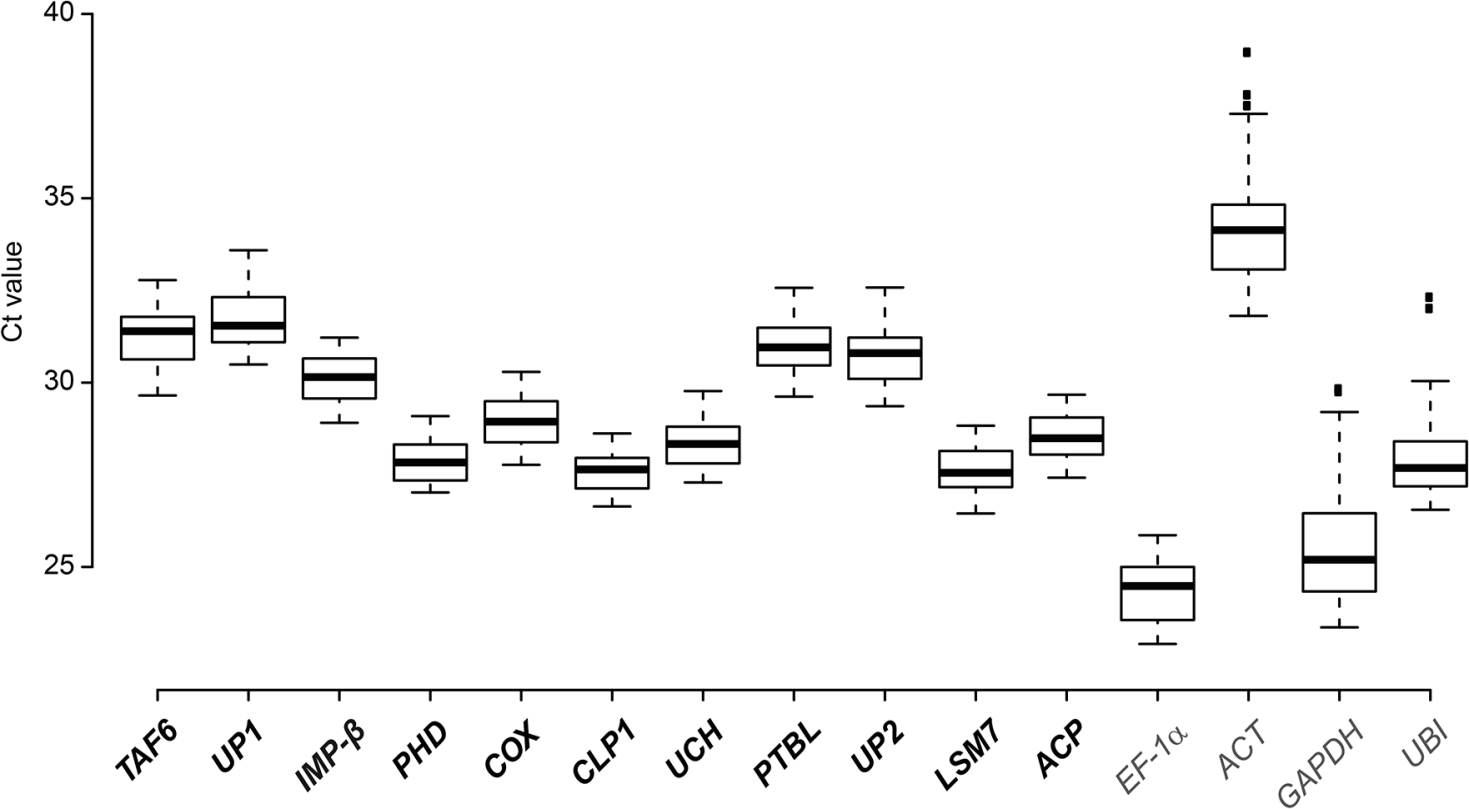
Expression profiles of new candidate reference genes and classical housekeeping genes from tomato. Box plot graphs of Ct values for each reference gene tested in all samples (n = 48). Ct values are inversely proportional to the amount of template. Boxes indicate the 25/75 percentiles, median values are represented by black lines. Whisker caps indicate the value range, dots represent outliers. New reference gene candidates are indicated in bold.

qRT-PCR analyses of the 11 most stably expressed genes (Table 1) were performed to validate their expression stability in *S*. *lycopersicum* cv. MM infected with *Xcv*. The genes encode a TFIID subunit (*TAF6*), importin β (*IMP-β*), a PHD finger family protein (*PHD*), a cytochrome c oxidase subunit (*COX*), polyribonucleotide 5´-hydroxyl-kinase Clp1 (*CLP1*), a ubiquitin carboxyl-terminal hydrolase family protein (*UCH*), a polypyrimidine tract-binding protein-like protein (*PTBL*), U6 snRNA-associated Sm-like protein LSm7 (*LSM7*) and an acyl carrier protein (*ACP*), as well as two unknown proteins (*UP1* and *UP2*; Table 1, S2 Table). For comparison, four housekeeping genes were analyzed that are widely used as references, namely *actin* (*ACT*), *EF-1α*, *GAPDH* and *ubiquitin* (*UBI*). First, suitability of oligonucleotides (S1 Table) and target sequences was confirmed. Melting curves and gel electrophoresis revealed unique amplicons for all oligonucleotide combinations used validating their specificity (S3 Fig). PCR efficiencies ranged between 80.48 and 99.71% (S1 Table). For expression analysis of the reference gene candidates, total RNA was isolated from tomato leaves 0, 6, 10 and 24 h after treatment with 10 mM MgCl_2_, *Xcv* 85–10, 85–10Δ*hrcN* and 85-10(p*avrBs4*), respectively. The latter strain induces the ETI, i.e., the HR in *S*. *lycopersicum* cv. MM due to the *Bs4*-dependent recognition of the avirulence protein AvrBs4, a member of the TAL effector family [66]. Technical duplicates of three biological replicates were subjected to qRT-PCR analysis. Average Ct (cycle threshold) values of the new reference gene candidates ranged from 27.1 (*CLP1*) to 31.1 (*UP1*; Fig 2). To select the optimal reference genes, we used three different algorithms to evaluate our qRT-PCR results: geNorm [58], NormFinder [59] and BestKeeper [60].

#### geNorm analysis

**Fig 3:**
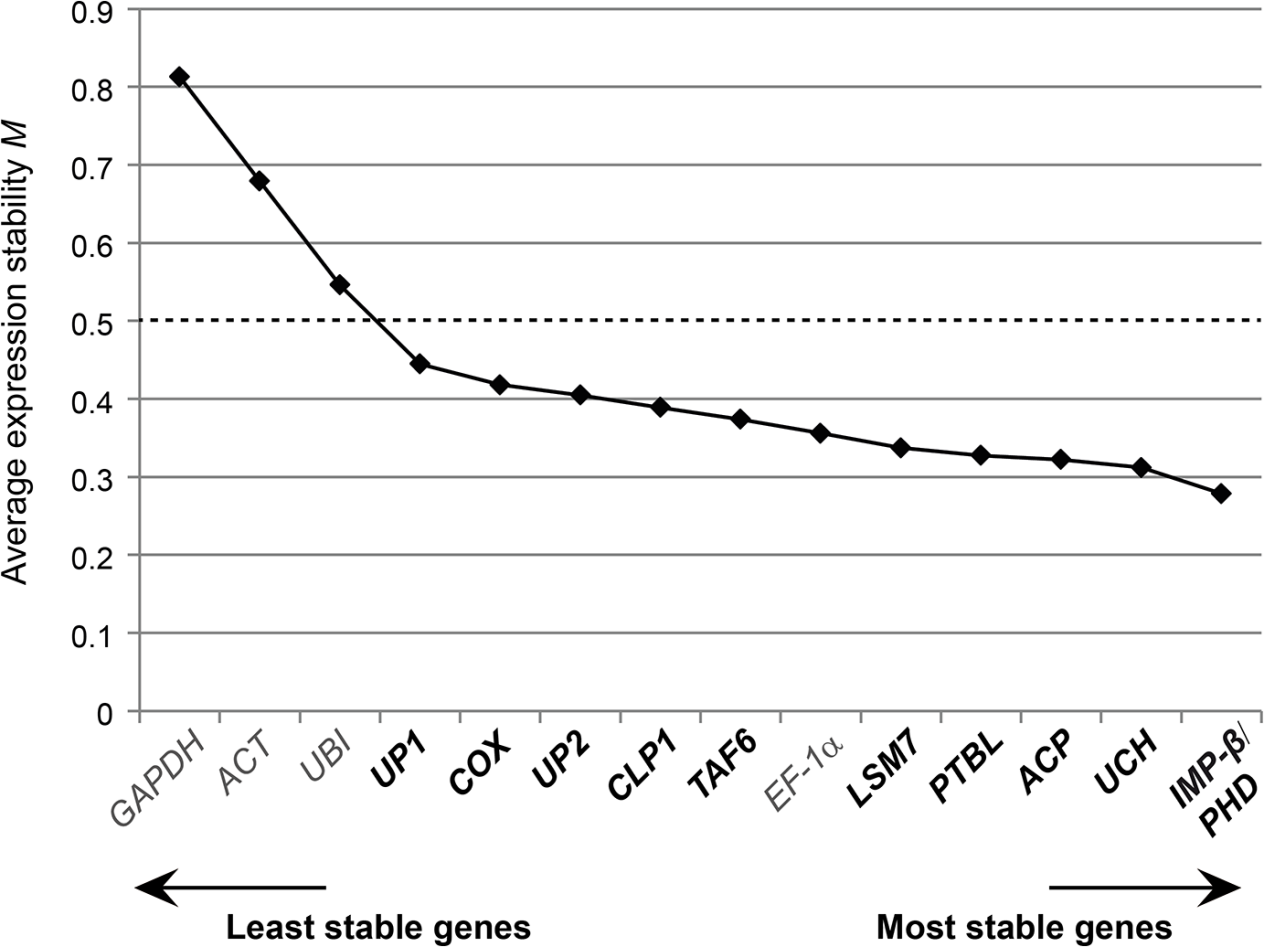
Expression stability of candidate reference genes in *Xcv*-infected and mock-treated tomato plants evaluated by geNorm. Tomato reference genes were ranked based on expression stability calculated by geNorm. New reference gene candidates are indicated in bold. *M* values represent the average expression stability of each gene (n = 48). The cut-off value for reliable reference genes is indicated by a dashed line.

The geNorm software provides a ranking of the tested genes based on a stability value *M* which is calculated by average pairwise variation of each candidate gene combination [58]. The lower the *M* value, the higher the expression stability of the gene. Eventually, the algorithm selects an optimal pair of reference genes out of the candidate set analyzed. Considering a cutoff of *M* ≤ 0.5, the traditional references *GAPDH*, *ACT* and *UBI* proved unreliable for the normalization of qRT-PCR data under the experimental conditions chosen (Fig 3). By contrast, all newly identified tomato candidate genes and *EF-1α* represent suitable references, with *IMP-β* and *PHD* being optimal (Fig 3).

#### NormFinder analysis

**Fig 4:**
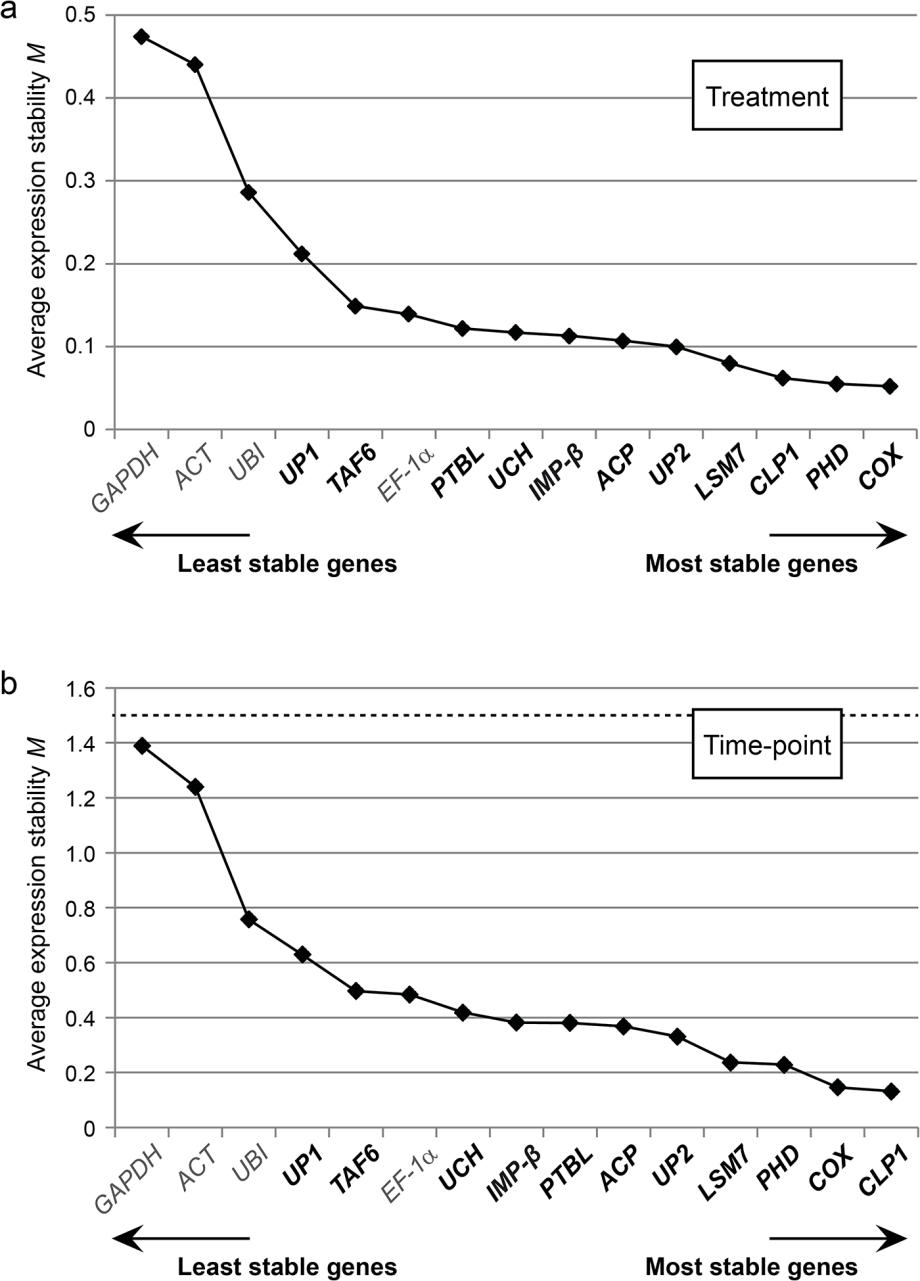
Expression stability of candidate reference genes in *Xcv*-infected and mock-treated tomato plants evaluated by NormFinder. Tomato reference genes were ranked based on expression stability calculated by NormFinder (n = 48). New reference gene candidates are indicated in bold. The cut-off value for reliable reference genes is indicated by a dashed line. Sample groups were defined based on (a) treatment [MgCl_2_, *Xcv* 85–10, 85–10Δ*hrcN* and 85-10(p*avrBs4*)] or (b) time-point of harvesting (0, 6, 10 and 24 hpi).

Next, we analyzed the qRT-PCR data using NormFinder [59]. The stability value *M* calculated by this “model-based variance estimation approach” considers not only the “overall expression variation” measured in different samples, but additionally takes into account variations among and inside sample subgroups [59]. Thus, the algorithm avoids co-regulated reference genes which display systematic intergroup variation and would lead to erroneous conclusions. Since we are interested in changes of plant gene expression levels induced by different *Xanthomonas* strains but also in expression level changes over a certain time period, two separate NormFinder analyses were performed with sample subgroups defined based on treatment [MgCl_2_, *Xcv* 85–10, 85–10Δ*hrcN* and 85-10(p*avrBs4*)] and time-point of sampling (0, 6, 10 and 24 hpi), respectively. As shown in Fig 4, all tested genes fulfill the minimal requirement for suitable reference genes, i.e., possess an *M* value below 1.5. However, the traditionally employed reference genes *ACT* and *GAPDH* were considerably less stable than the other genes, whereas *EF-1α* and *UBI* seemed more suitable under the chosen experimental conditions. The top-ranked references, however, were among the newly identified candidate genes, namely *COX* > *PHD* > *CLP1* > *LSM7* with respect to the grouping by treatment (Fig 4A).

#### BestKeeper analysis

We compared the six most stable new reference genes according to NormFinder with the four classical reference genes using BestKeeper [60]. This tool evaluates the suitability of up to 10 reference genes based on the calculation of Ct value variations, performing pair-wise correlations of all candidate gene combinations. Extreme samples (x-fold over-/under-expression) are also considered. As shown in Table 2, expression of all genes except for *ACT* and *GAPDH* fluctuated in a range compatible with standard deviations (SD) [± Ct] < 1 and SD [± x-fold] < 2, which represents an acceptable overall variation [60]. Notably, BestKeeper evaluated all six new reference gene candidates as better suited than the four traditional housekeeping genes, with *PHD* > *CLP1* > *LSM7* > *COX* being the top four. Taken together, regardless of the ranking order, geNorm, NormFinder and BestKeeper evidenced the superior expression stability of the new tomato reference genes under the experimental conditions chosen.

**Table 2:** Descriptive statistics of six newly identified and four classical tomato reference genes based on their crossing point values in all samples combined (n = 48) as calculated by BestKeeper.

Ranking	1	2	3	4	5	6	7	8	9	10
Gene name^a)^	*CLP1*	*PHD*	*LSM7*	*ACP*	*IMP-β*	*COX*	*EF-1a*	*UBI*	*ACT*	*GAPDH*
Geo Mean [Ct]	27.58	27.87	27.62	28.51	30.11	28.94	24.32	27.95	34.16	25.57
Min [Ct]	26.64	27.02	26.45	27.42	28.91	27.77	22.91	26.55	31.81	23.36
Max [Ct]	28.62	29.09	28.83	29.67	31.22	30.29	25.86	32.32	38.96	29.83
SD [± Ct]	0.41	0.47	0.53	0.53	0.55	0.61	0.72	0.86	1.27	1.41
CV [% Ct]	1.50	1.67	1.90	1.86	1.83	2.09	2.97	3.09	3.70	5.50
Min [x-fold]	-1.88	-1.78	-2.16	-2.02	-2.06	-1.75	-2.52	-2.61	-3.33	-3.66
Max [x-fold]	2.00	2.29	2.22	2.10	1.96	1.92	2.76	20.28	11.75	12.24
SD [± x-fold]	1.28	1.32	1.37	1.38	1.39	1.44	1.55	1.69	2.15	2.34

^a)^ New reference gene candidates are indicated in bold. [Ct], cycle threshold; Geo Mean [Ct], geometric mean of Ct; Min [Ct] and Max [Ct], the extreme values of Ct; SD [± Ct], standard deviation of the Ct; CV [% Ct], CV expressed as a percentage on the Ct level; Min [x-fold] and Max [x-fold], the extreme values of expression levels expressed as an absolute x-fold over- or under-regulation coefficient; SD [± x-fold], standard deviation of the absolute regulation coefficients.

### Quantification of immunity marker genes in infected tomato leaves

**Fig 5:**
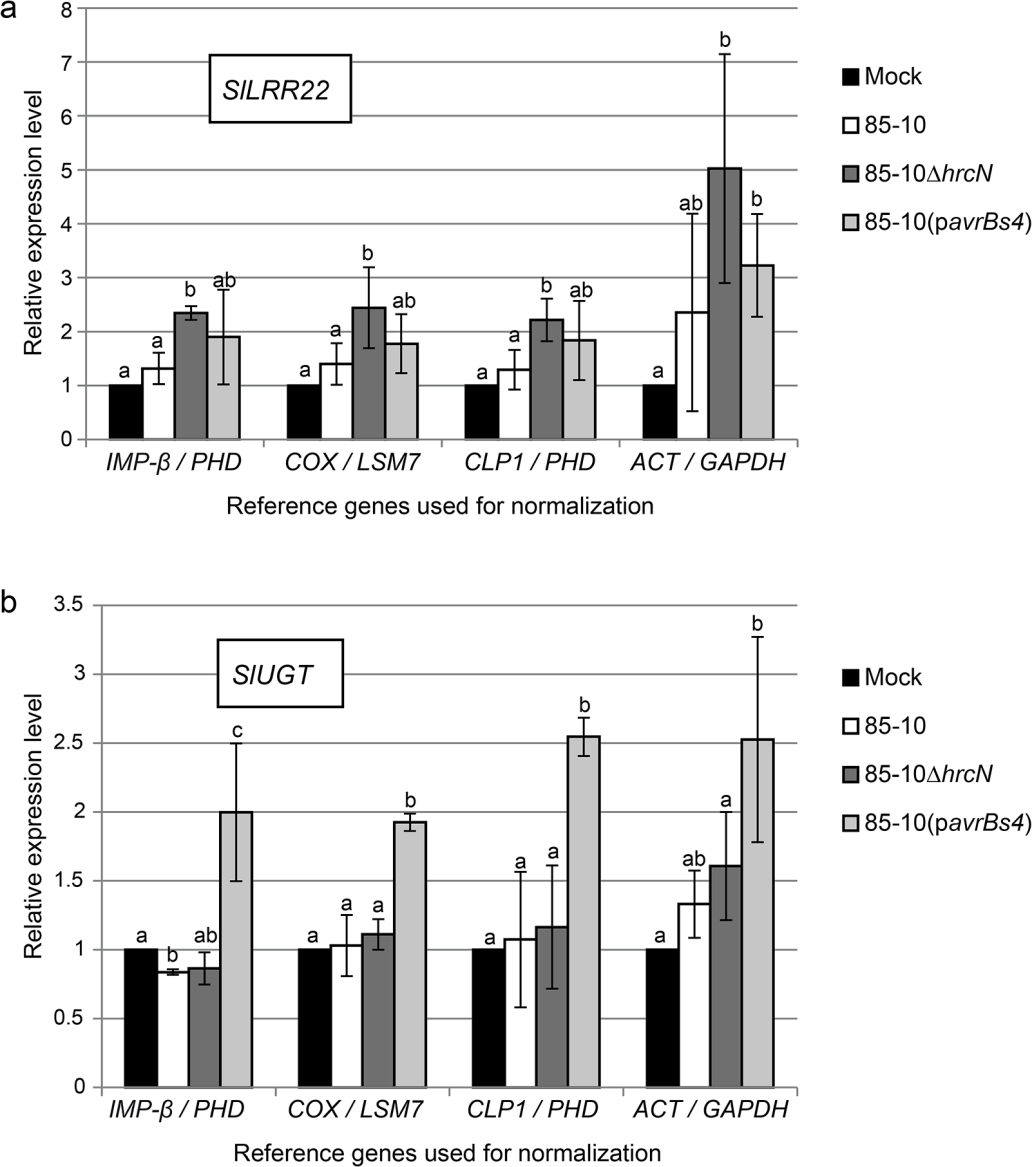
Relative expression of PTI and ETI marker genes in *Xcv*-infected and mock-treated tomato plants. Expression patterns of *SlLRR22* (a) and *SlUGT* (b) in *S*. *lycopersicum* cv. MM leaves treated with 10 mM MgCl_2_ (mock) or 5×10^8^ cfu/ml of *Xcv* 85–10, 85–10Δ*hrcN* and 85-10(p*avrBs4*), respectively, 6 hpi. qRT-PCR data were normalized with different reference gene pairs. Values are mean-fold changes in mRNA levels in *Xcv*-infected relative to mock-inoculated leaves for three biological replicates. Error bars indicate standard deviation (SD). Letters denote statistically significant differences (Student´s *t*-test, *P* < 0.05).

We applied our findings to the analysis of two target genes previously reported to be induced during PTI and ETI, respectively, *LRR22* [67] and an UDP-glucosyltransferase gene (*UGT*, Solyc09g092500 [68]). For this, total RNA was analyzed from tomato leaves six hours after treatment with 10 mM MgCl_2_, *Xcv* 85–10, 85–10Δ*hrcN* and 85-10(p*avrBs4*), respectively. To increase the accuracy of normalization we took into account two reference genes. We compared the two best reference genes identified by geNorm (*IMP-β* and *PHD*), NormFinder (*PHD* and *COX*) and BestKeeper (*CLP1* and *PHD*) with the two least-stable genes, *GAPDH* and *ACT*, for their ability to provide reliable relative quantification of *SlLRR22* and *SlUGT* by qRT-PCR. As shown in Fig 5, accumulation of *SlLRR22* transcript was approximately two-fold higher in the leaves treated with 85–10Δ*hrcN* than in the mock control if compared to any of the new reference gene combinations. By contrast, comparison to the suboptimal references revealed an apparent five-fold induction of gene expression. In addition, referring to *ACT* and *GAPDH* suggested a more than two-fold upregulation of *SlLRR22* by the *Xcv* WT strain 85–10 and by 85-10(p*avrBs4*), the latter induction being significant, which was not detectable with any of the superior reference genes. Notably, standard deviations between the different biological datasets were substantially lower if one of the new reference gene combinations was employed. The analysis of the ETI marker gene, *SlUGT*, did not show pronounced differences in the expression pattern depending on the reference genes chosen. In all cases, transcript abundance was significantly higher in the leaves treated with the avirulent strain 85-10(p*avrBs4*) than in the mock-infiltrated leaves. However, a slight induction of *SlUGT* expression by both *Xcv* 85–10 and 85–10Δ*hrcN* was only detected when the traditional references were employed. A possible explanation for these results is downregulation of *ACT* and/or *GAPDH* by *Xcv* infection. To test this possibility, the expression of both genes was analyzed using the newly identified reference genes as normalization controls. As shown in S4 Fig, *GAPDH* transcript levels were indeed significantly lower in the leaf material inoculated with bacteria compared to the mock control, whereas *ACT* appeared not to be changed under these conditions.

### Selection and validation of pepper reference genes based on tomato orthologs

**Fig 6:**
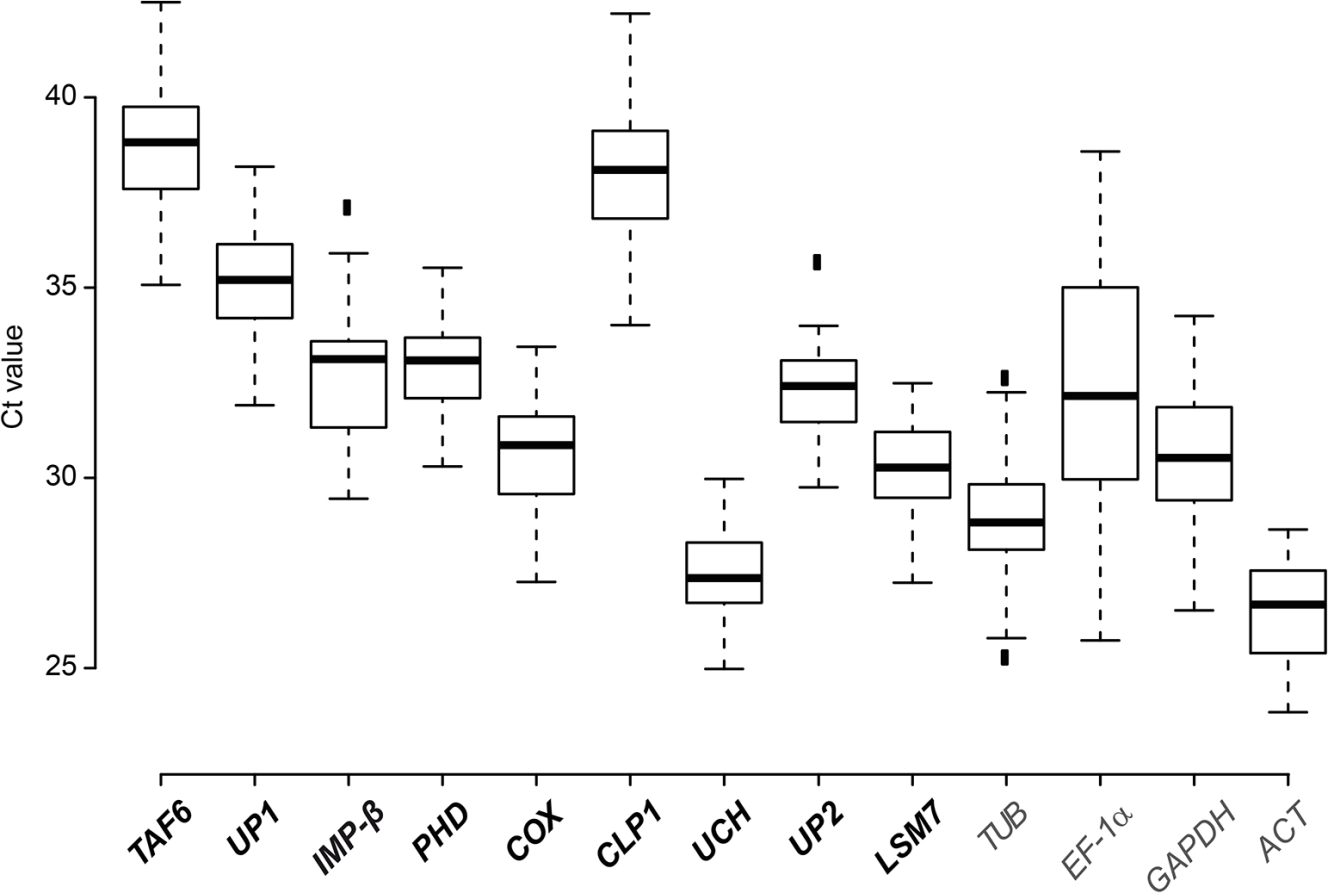
Expression profiles of new candidate reference genes and classical housekeeping genes from pepper. Box plot graphs of Ct values for each reference gene tested in all samples (n = 48). Ct values are inversely proportional to the amount of template. The boxes indicate the 25/75 percentiles, median values are represented by black lines. Whisker caps indicate the value range, dots represent outliers. New reference gene candidates are indicated in bold.

Based on the tomato microarray data, pepper orthologs of the eleven most stably expressed genes (Table 1) were identified by BLASTx against the European Nucleotide Archive (http://www.ebi.ac.uk/ena). Oligonucleotides for qRT-PCR were derived (S1 Table), and melting curve analysis and gel electrophoresis confirmed specific products for nine candidate genes (S5 Fig). PCR efficiencies ranged between 72.09 and 99.32% (S1 Table). For expression analysis, pepper ECW-30R (*Bs3*) leaves were infiltrated with 10 mM MgCl_2_, *Xcv* 85–10, 85–10Δ*hrcN* and 85-10(p*avrBs3*), respectively, and leaf material was harvested at 0, 6, 10 and 24 hpi. 85-10(p*avrBs3*) translocates the effector AvrBs3 which induces the HR in *Bs3* pepper plants. Technical duplicates of three biological replicates were subjected to qRT-PCR analysis. Average Ct values of the new reference gene candidates ranged from 27.4 (*UCH*) to 38.8 (*TAF6*; Fig 6). For comparison, the four classical reference genes *EF-1α*, *GAPDH*, *ACT* and *β*-*tubulin* (*TUB*) were also analyzed.

**Fig 7:**
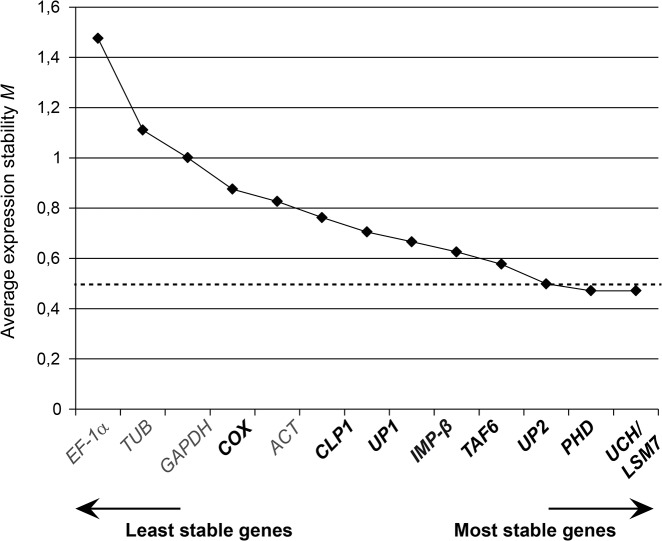
Expression stability of candidate reference genes in *Xcv*-infected and mock-treated pepper plants evaluated by geNorm. Ranking of *C*. *annuum* reference genes based on expression stability calculated by geNorm. New reference gene candidates are indicated in bold. *M* values represent the average expression stability of each gene (n = 48). The cut-off value for reliable reference genes is indicated by a dashed line.

The data were evaluated similarly to the analysis of the tomato reference genes described above. GeNorm analysis revealed that only three genes, *UCH*, *LSM7* and *PHD*, match the cutoff-value for a reliable reference gene (*M* ≤ 0.5). In general, the pepper orthologs of the newly identified tomato reference genes were more stably expressed than the traditional pepper references (Fig 7).

**Fig 8:**
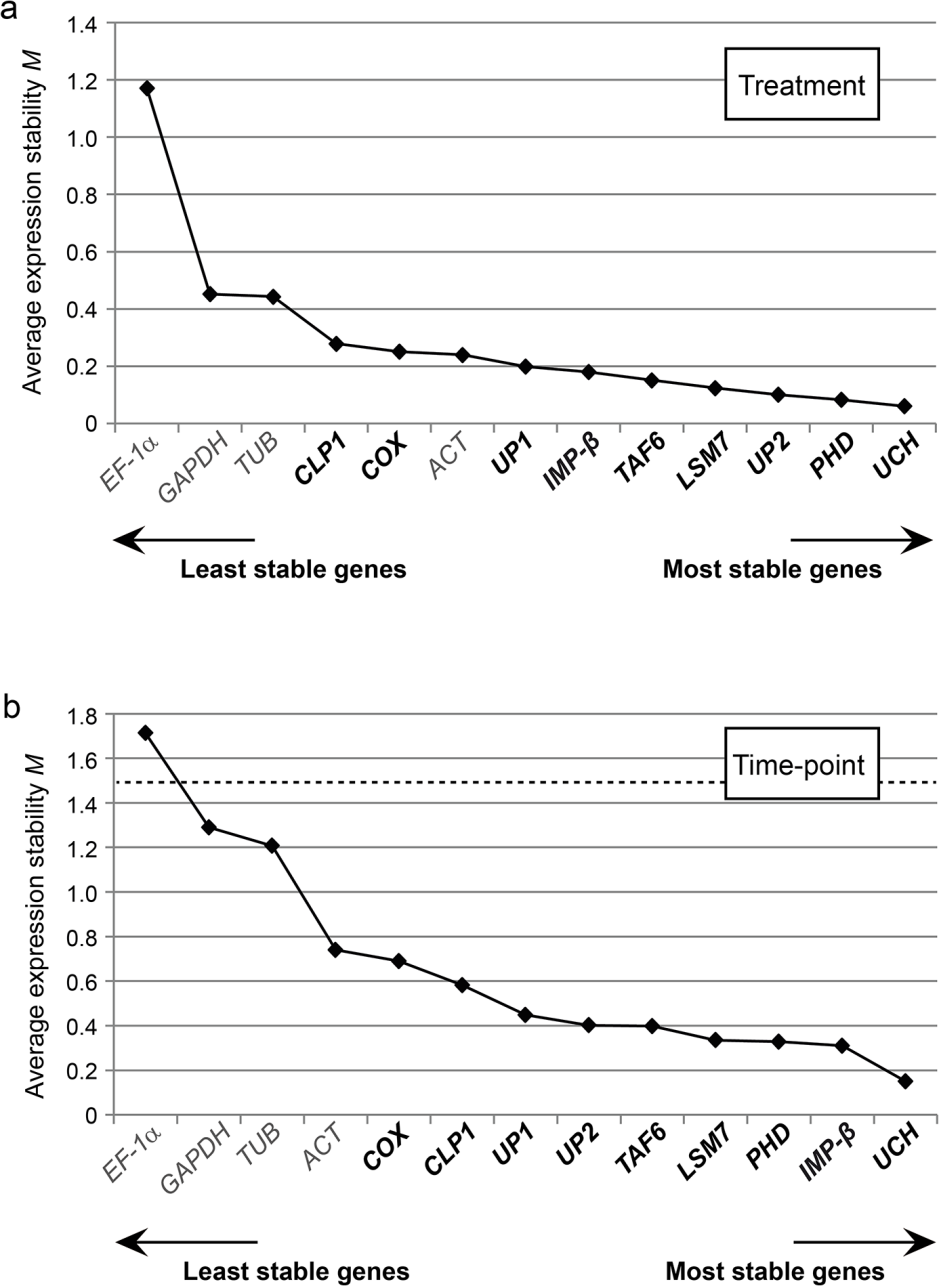
Expression stability of candidate reference genes in *Xcv*-infected and mock-treated pepper plants evaluated by NormFinder. Ranking of *C*. *annuum* reference genes based on expression stability calculated by NormFinder (n = 48). New reference gene candidates are indicated in bold. The cut-off value for reliable reference genes is indicated by a dashed line. Sample groups were defined based on (a) treatment [MgCl_2_, *Xcv* 85–10, 85–10Δ*hrcN* and 85-10(p*avrBs3*)] and (b) time-points of harvesting (0, 6, 10 and 24 hpi).

Using NormFinder, the classical reference gene *EF-1α* matched the requirements of a suitable reference gene (*M* < 1.5) when the sample subgroups were defined by treatment (Fig 8A), but turned out to be completely unreliable when the classification was based on the time-point of sampling (Fig 8B). *GAPDH* and *TUB* matched the minimal requirements of a reliable reference gene but were considerably less stable than the other genes tested, while *ACT* appeared more suitable. Notably, all newly identified reference genes were evaluated as reliable normalization controls with *UCH* > *PHD* > *UP2* > *LSM7* as the top-four when the grouping was based on treatment (Fig 8A).

BestKeeper analysis of the six best new pepper reference genes according to NormFinder and the four classical references surprisingly revealed that only one gene, *UCH*, fulfilled both requirements for a suitable normalization control in qRT-PCR studies, i.e., SD [± Ct] < 1 and SD [± x-fold] < 2 (Table 3). Most of the other genes matched at least the threshold for SD [± x-fold], whereas *EF-1α* appeared to be completely unreliable as reference gene (Table 3).

**Table 3:** Descriptive statistics of six newly identified and four classical pepper reference genes based on their crossing point values in all samples combined (n = 48) as calculated by BestKeeper.

Ranking	1	2	3	4	5	6	7	8	9	10
Gene name^a)^	*UCH*	*ACT*	*UP2*	*PHD*	*LSM7*	*TAF6*	*IMP-β*	*TUB*	*GAPDH*	*EF-1α*
Geo Mean [Ct]	27,33	26,52	32,18	32,90	30,05	38,62	32,71	28,95	30,46	31,97
Min [Ct]	24,98	23,84	29,76	30,31	27,25	35,09	29,46	25,28	26,52	25,73
Max [Ct]	29,98	28,65	35,68	35,54	32,50	42,53	37,12	32,64	34,27	38,60
SD [± Ct]	0,95	1,00	1,01	1,04	1,20	1,24	1,31	1,34	1,53	2,81
CV [% Ct]	3,48	3,75	3,14	3,17	4,00	3,21	4,00	4,61	5,00	8,73
Min [x-fold]	-4,62	-5,27	-4,09	-4,27	-4,88	-3,62	-5,18	-9,39	-10,14	-48,01
Max [x-fold]	5,64	3,75	7,68	4,37	3,99	4,16	9,37	9,47	9,39	61,27
SD [± x-fold]	1,42	1,44	1,45	1,46	1,55	1,57	1,61	1,63	1,74	2,78

^a)^ New reference gene candidates are indicated in bold. [Ct], cycle threshold; Geo Mean [Ct], geometric mean of Ct; Min [Ct] and Max [Ct], the extreme values of Ct; SD [± Ct], standard deviation of the Ct; CV [% Ct], CV expressed as a percentage on the Ct level; Min [x-fold] and Max [x-fold], the extreme values of expression levels expressed as an absolute x-fold over- or under-regulation coefficient; SD [± x-fold], standard deviation of the absolute regulation coefficients.

### Quantification of PTI and ETI marker genes in infected pepper leaves

**Fig 9:**
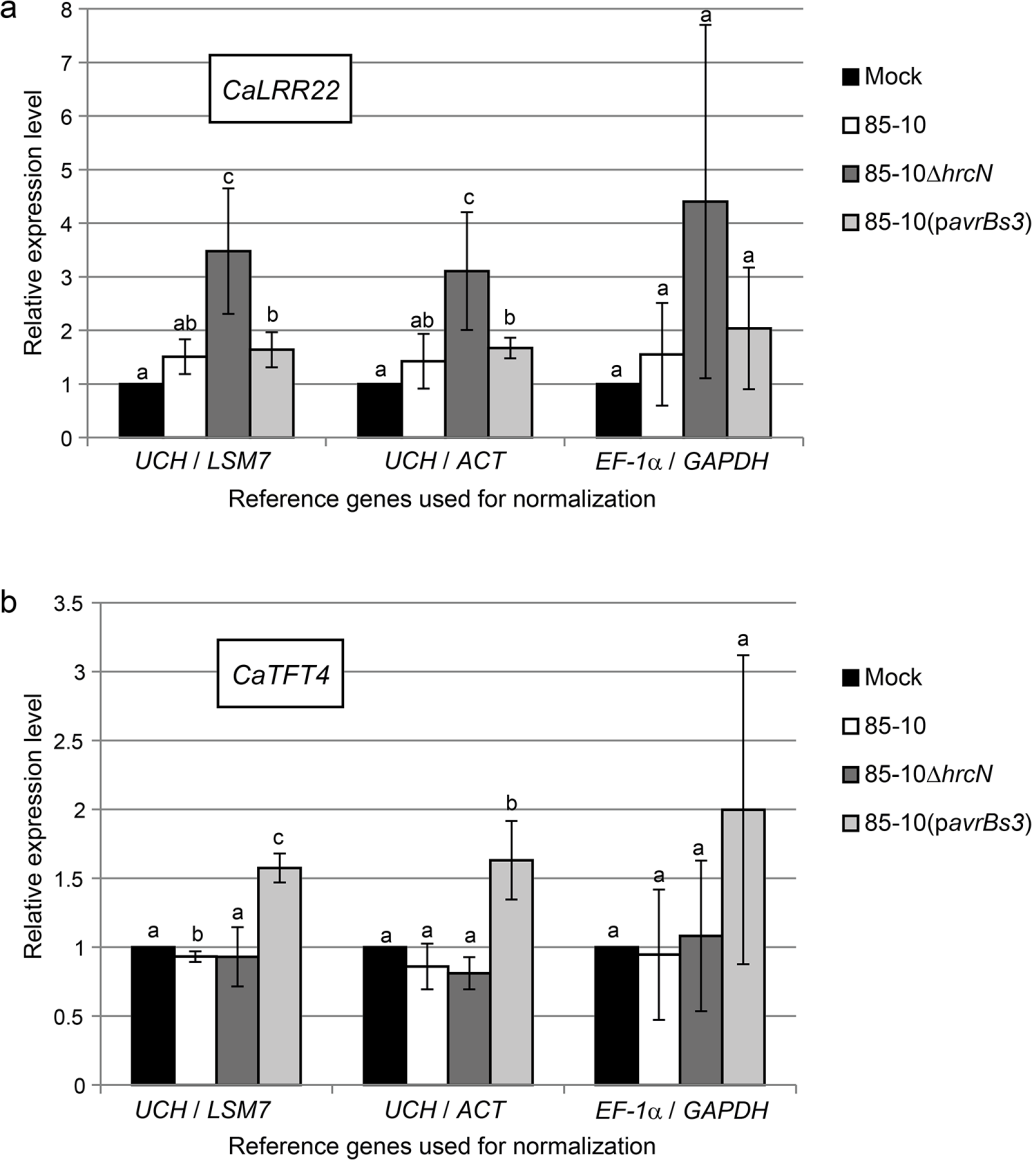
Relative expression of PTI and ETI marker genes in *Xcv*-infected and mock-treated pepper leaves. Expression patterns of *CaLRR22* (a) and *CaTFT4* (b) in *C*. *annuum* ECW-30R leaves treated with 10 mM MgCl_2_ (mock) or 5×10^8^ cfu/ml of *Xcv* 85–10, 85–10Δ*hrcN* and 85-10(p*avrBs3*), respectively, six hpi. qRT-PCR data were normalized with different reference gene pairs. Values are mean fold changes in mRNA levels in *Xcv*-infected relative to mock-inoculated leaves for three biological replicates. Error bars indicate SD. Letters denote statistically significant differences (Student´s *t*-test, *P* < 0.05).

We compared the two best reference genes from pepper identified by geNorm, i.e., *UCH* and *LSM7* with the best reference genes according to BestKeeper, *UCH* and *ACT*, and the traditional reference genes *EF-1α* and *GAPDH* for their ability to provide reliable relative quantification of the target genes *LRR22* and *TFT4*, which are induced during PTI and ETI, respectively [67, 69]. As shown in Fig 9, employment of different reference genes did not result in substantial differences in the expression patterns of *CaLRR22* and *CaTFT4*. The T3S-deficient *Xcv* strain 85–10Δ*hrcN* led to significantly higher expression of *CaLRR22* compared to the WT strain 85–10 and *Xcv* 85-10(p*avrBs3*). *CaTFT4* was induced significantly during the incompatible interaction with *Xcv* 85-10(p*avrBs3*), similarly to the reported induction after recognition of the type III effector AvrBs2 [69]. However, the observed differences in target gene expression levels were only judged as significant when the newly identified reference genes were used, but not with the traditional combination *EF-1α*/*GAPDH*. Utilization of the newly identified normalization controls resulted in significantly lower standard deviations underlining the higher reproducibility of the results in different experiments (Fig 9).

## Discussion

Correct normalization of gene transcripts depends on the choice of suitable reference genes. This is essential for reliable analyses of gene expression by qRT-PCR and has to be established for specific experimental conditions [4]. Based on microarray expression analyses of >34,000 genes, we identified and validated 11 novel tomato reference genes with superior expression stability under biotic stress conditions, i.e., challenge by the bacterial pathogen *Xcv*. Although the new reference genes do not comprise “classical” housekeeping genes, homologies on the protein level indicate putative roles in basic cell functions, e.g., oxidation/reduction processes (*COX*), mRNA processing (*LSM7*, *CLP1*, *PTBL*), regulation of transcription/chromatin dynamics (*PHD*), nuclear import (*IMP-β*) and fatty acid biosynthesis (*ACP*; S2 Table). The three statistical programs we used for the evaluation of gene expression stability, geNorm, NormFinder and BestKeeper, slightly differed in the ranking of the reference gene candidates, which was also observed in previous studies and is probably due to different algorithms underlying the programs [23, 30, 31, 70]. Importantly, the newly identified genes were usually evaluated as more stable than the traditional housekeeping genes we analyzed for comparison and, notably, always included the optimal normalization control identified by the respective program. Based on our results, we recommend the use of *PHD* and *LSM7* as reference genes for normalization in future plant gene expression studies in the *Xcv*-tomato pathosystem.

To the best of our knowledge, previous studies of pepper and tomato comparing reference gene stabilities selected candidates solely based on homology. It was shown that different genes, often housekeeping genes, are preferable under different conditions [23, 30, 38, 62, 71–73]. Notably, our microarray data revealed that the expression of classical tomato housekeeping genes varied considerably, confirmed by qRT-PCR studies of selected genes. In particular, *GAPDH* and *ACT* were attested a variability too high for a reliable reference gene by geNorm and BestKeeper, respectively. Therefore, we do not recommend the further employment of these genes as normalization controls in qRT-PCR analysis of tomato genes after pathogen infection, especially because we clearly showed an *Xcv*-dependent downregulation of *GAPDH* expression. Taken together, our results demonstrate the advantage of an unbiased, whole transcriptome-based approach to identify suitable reference genes. Concordantly, several whole-transcriptome analyses of different plant species and experimental setups identified other than traditional housekeeping genes as the most stably expressed genes [37–43].

It is, however, not feasible to perform microarray analyses for reference gene identification every time the experimental setup is changed. Therefore, one has to resort also to the homology-based selection of candidate genes. The identification of suitable candidates can be strongly improved by using orthologs of genes that were experimentally verified as appropriate references in related organisms under similar experimental or developmental conditions [38, 74–76]. We used such an approach to identify the pepper orthologs of our new superior tomato reference genes and determined *UCH* and *PHD* as the most suitable references for normalization of plant gene expression in the *Xcv*-pepper pathosystem. Interestingly, one of the traditional reference genes, *ACT*, also turned out to be stably expressed in our experimental setup. This contradicts the results of Wan et al. who described *ACT* as relatively unstable under different abiotic stresses and hormonal treatments [72]. On the other hand, *EF-1α* turned out to be the most unstable pepper gene in our analyses although it was published as one of the least-variably expressed genes under abiotic stress conditions and hormone treatment [71]. This underpins the observation that a chosen gene can be stable under certain conditions but highly variable under others [3]. It should be noted that differences between the pepper lines used in the different studies might also play a role.

Although our selection of pepper orthologs of the new tomato reference genes surely represents an improvement compared to the selection of genes based on their known or suspected housekeeping roles, the ranking of our tomato reference genes and their pepper equivalents illustrates that the expression of gene orthologs can distinctly differ even between related plant species. In general, the *M* values calculated by NormFinder were lower for the tomato genes compared with their pepper orthologs. This difference appeared even more pronounced using geNorm which judged only three of the pepper genes tested as reliable reference genes. Similarly, using Bestkeeper, only one pepper gene, *UCH*, matched both requirements for a suitable reference gene. Therefore, we would like to emphasize that, even if our new pepper reference genes proved to be superior to most of the classical normalization controls we analyzed, a whole-transcriptome analysis of *Xcv*-challenged pepper plants might uncover even more suitable reference genes.

Taken together, the newly discovered tomato reference genes proved to be superior normalization controls for qRT-PCR studies of *Xcv*-infected tomato plants. In addition, they led to successful identification of the pepper orthologs as reliable reference genes in qRT-PCR analyses of the *Xcv*-pepper pathosystem. Similarly, these genes might be useful for the identification of suitable qRT-PCR normalization controls in other plant species for the analysis of plant gene expression during pathogen infection.

## Supporting Information

S1 Fig Experimental setup and data cluster analysis of the tomato microarray screens. (a) First microarray experiment. 12 plants were inoculated with *Xcv* strains 85–10 and 85–10Δ*hrcN*, four leaves per plant. Leaf material was harvested 45 min post infiltration (mpi) and 6, 10 and 24 hpi and pooled (four plants each). RNA was isolated, and the cDNAs used for microarray hybridizations. (b) Second microarray experiment. Three separate infiltrations of four plants each were performed with 10 mM MgCl_2_ (mock) and *Xcv* 85–10Δ*hrcN*. Leaf material was harvested 0, 4, 8 and 16 hpi and analyzed as described in (a). Dendrograms on the right show hierarchical cluster analysis of the respective microarray dataset (normalized log-expression values).
(TIF)

S2 Fig Functional classification of the 50 most stable reference genes in *Xcv*-infected versus uninfected tomato plants. Functional categories of the 50 most stably expressed tomato genes according to microarray hybridization data, based on Gene Ontology (GO) terms of the respective *A*. *thaliana* orthologs.
(TIF)

S3 Fig Validation of oligonucleotide pairs of new tomato reference gene candidates for qRT-PCR analysis. Presence of unique amplicons as a measure of PCR amplification specificity was determined (a) by electrophoresis on 1% agarose gel and (b) by melting curve analysis.
(TIF)

S4 Fig Relative expression of *ACT* and *GAPDH* in *Xcv*-infected and mock-treated tomato plants. Expression patterns of (a) *SlACT* and (b) *SlGAPDH* in *S*. *lycopersicum* cv. MM leaves 6 hpi of 10 mM MgCl_2_ (mock) or 5×10^8^ cfu/ml of *Xcv* 85–10, 85–10Δ*hrcN* and 85-10(p*avrBs4*), respectively. qRT-PCR data were normalized with different reference gene pairs. Values are mean-fold changes in mRNA levels in *Xcv*-infected relative to mock-inoculated leaves for three biological replicates. Error bars indicate SD. Letters denote statistically significant differences (Student´s *t*-test, *P* < 0.05).
(TIF)

S5 Fig Validation of oligonucleotide pairs of new pepper reference gene candidates for qRT-PCR analysis. Presence of unique amplicons as a measure of PCR amplification specificity was determined (a) by electrophoresis on a 1% agarose gel and (b) by melting curve analysis.
(TIF)

S1 Table Oligonucleotide sequences used for qRT-PCR analyses. (DOC)

S2 Table Functional classification of Arabidopsis orthologs corresponding to the new tomato reference genes. (DOC)
