# Supplementary figures and images for: Genome-Wide Identification and Validation of Reference Genes in Infected Tomato Leaves for Quantitative RT-PCR Analyses
Source: PLoS One. 2015 Aug 27;10(8):e0136499. doi: 10.1371/journal.pone.0136499 (PMC4552032; doi:10.1371/journal.pone.0136499)

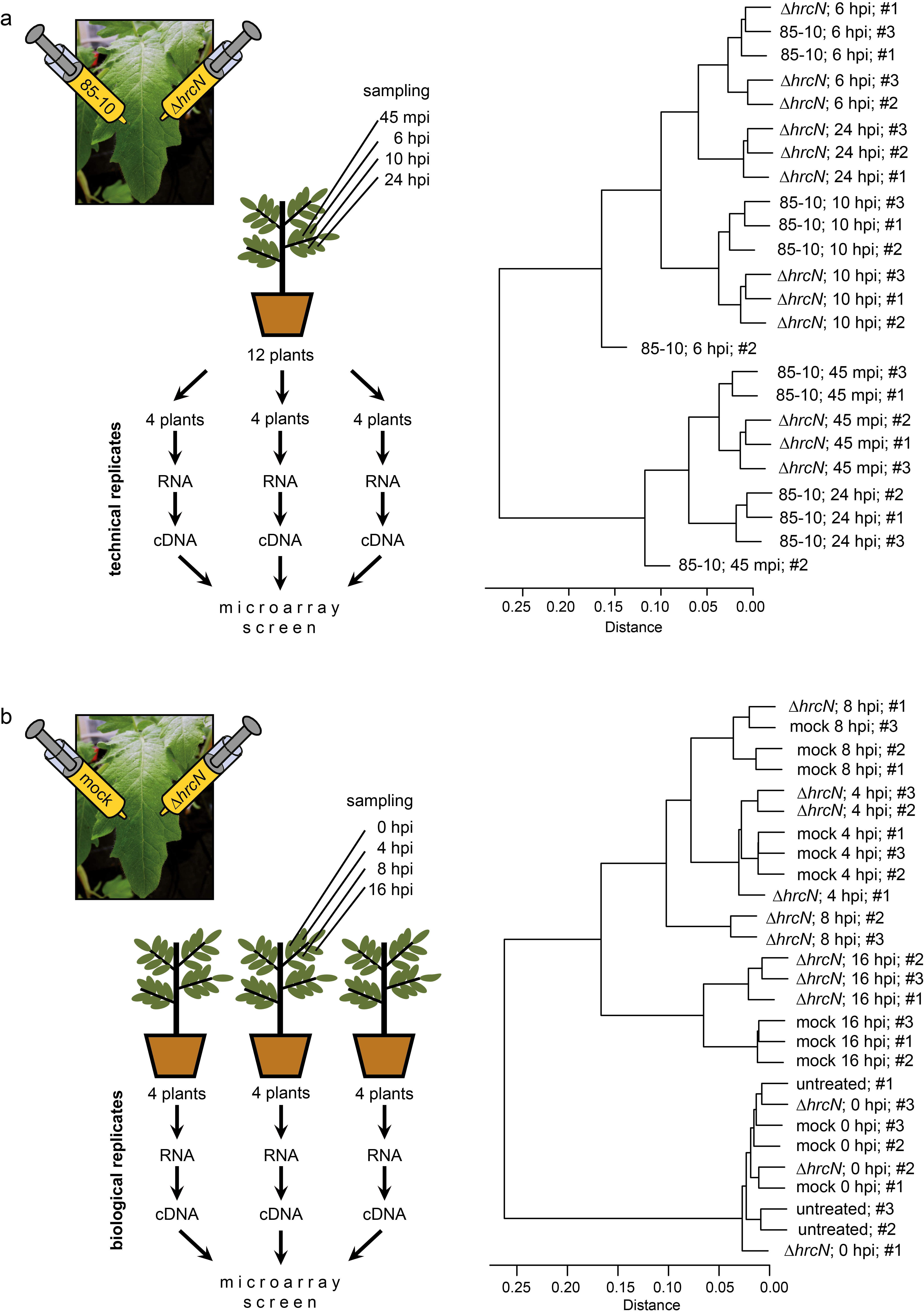

Supplement: S1 Fig — (a) First microarray experiment. 12 plants were inoculated with Xcv strains 85–10 and 85–10ΔhrcN, four leaves per plant. Leaf material was harvested 45 min post infiltration (mpi) and 6, 10 and 24 hpi and pooled (four plants each). RNA was isolated, and the cDNAs used for microarray hybridizations. (b) Second microarray experiment. Three separate infiltrations of four plants each were performed with 10 mM MgCl2 (mock) and Xcv 85–10ΔhrcN. Leaf material was harvested 0, 4, 8 and 16 hpi and analyzed as described in (a). Dendrograms on the right show hierarchical cluster analysis of the respective microarray dataset (normalized log-expression values). (TIF) [file pone.0136499.s001.tif]

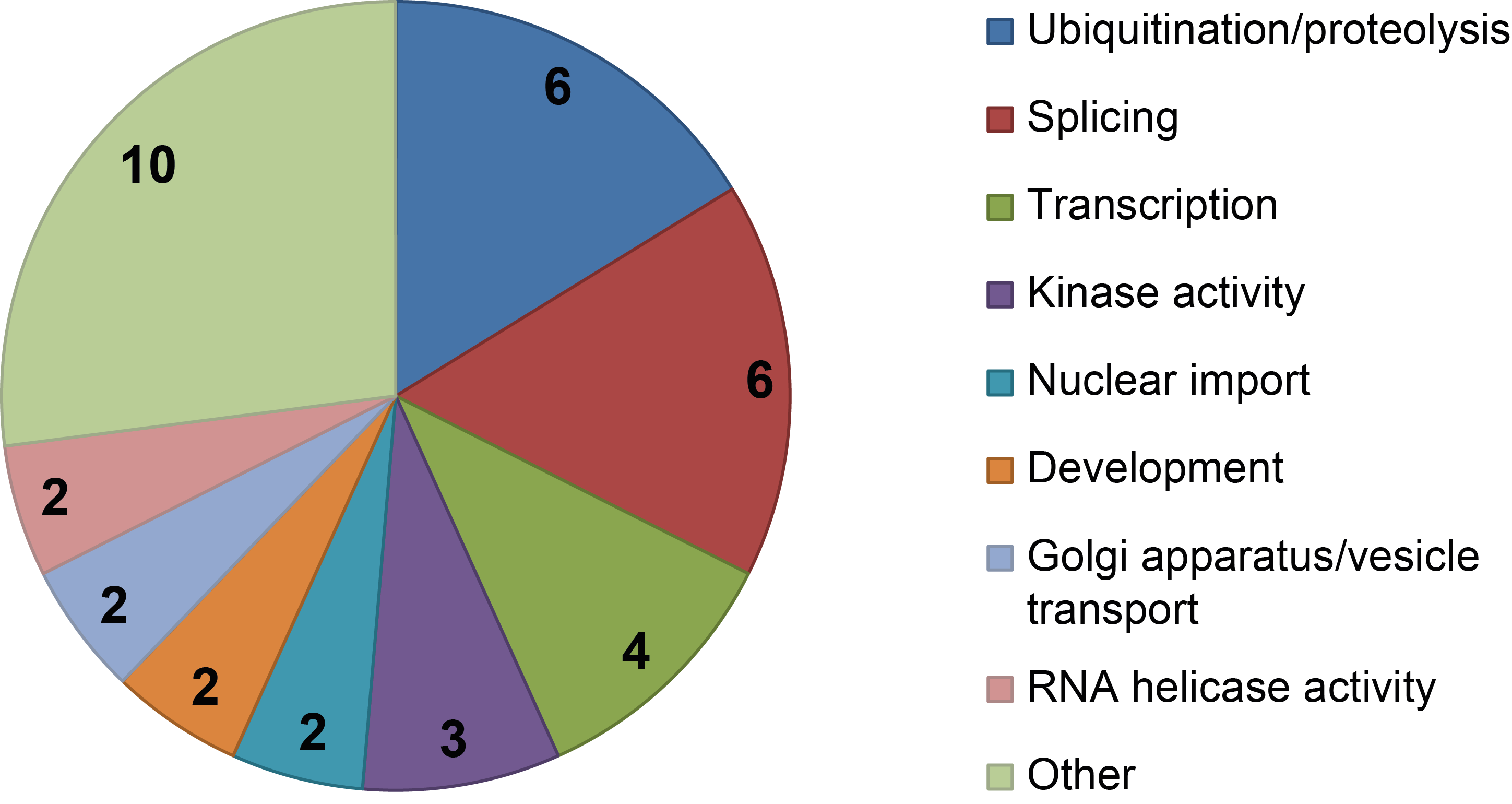

Supplement: S2 Fig — Functional categories of the 50 most stably expressed tomato genes according to microarray hybridization data, based on Gene Ontology (GO) terms of the respective A. thaliana orthologs. (TIF) [file pone.0136499.s002.tif]

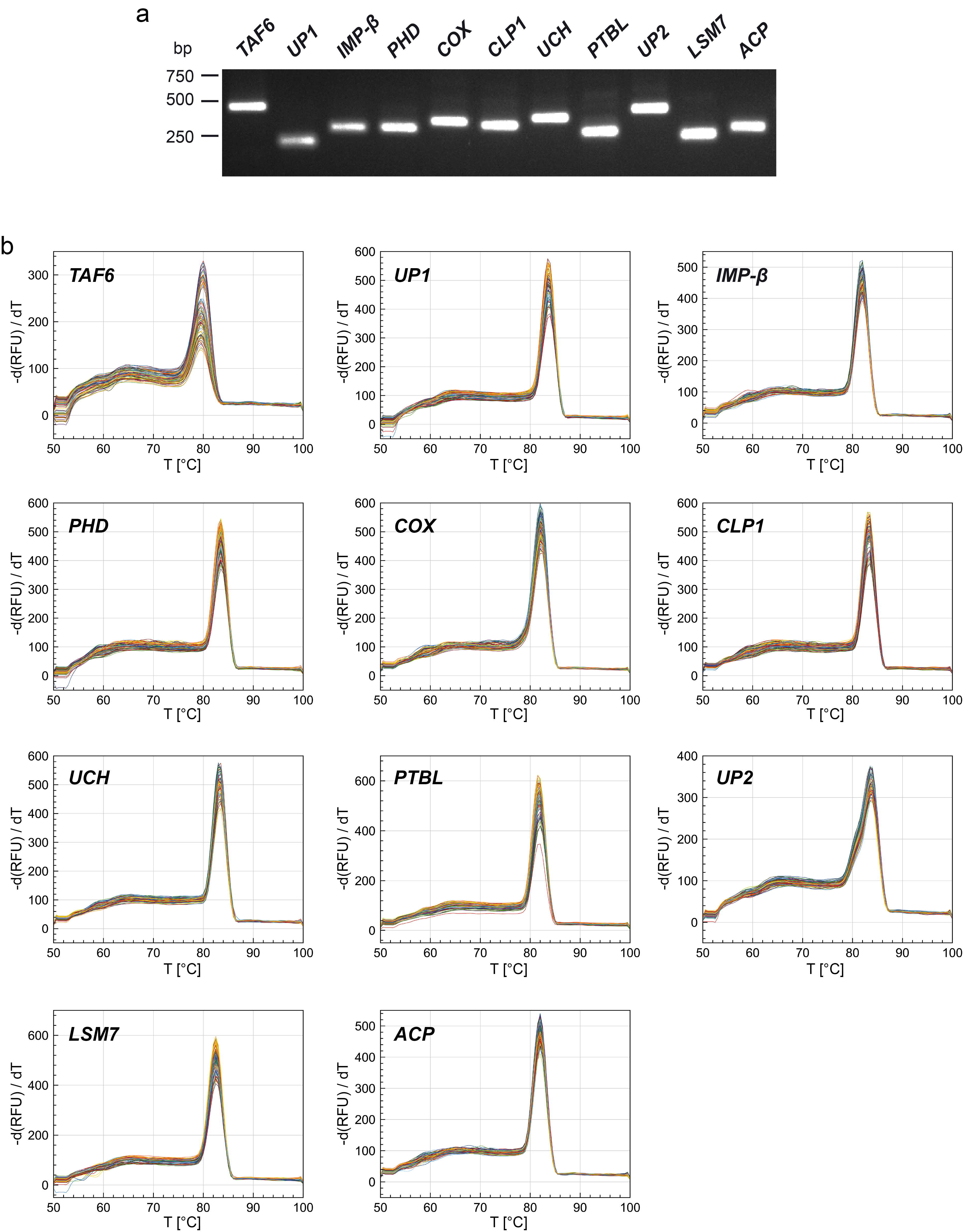

Supplement: S3 Fig — Presence of unique amplicons as a measure of PCR amplification specificity was determined (a) by electrophoresis on 1% agarose gel and (b) by melting curve analysis. (TIF) [file pone.0136499.s003.tif]

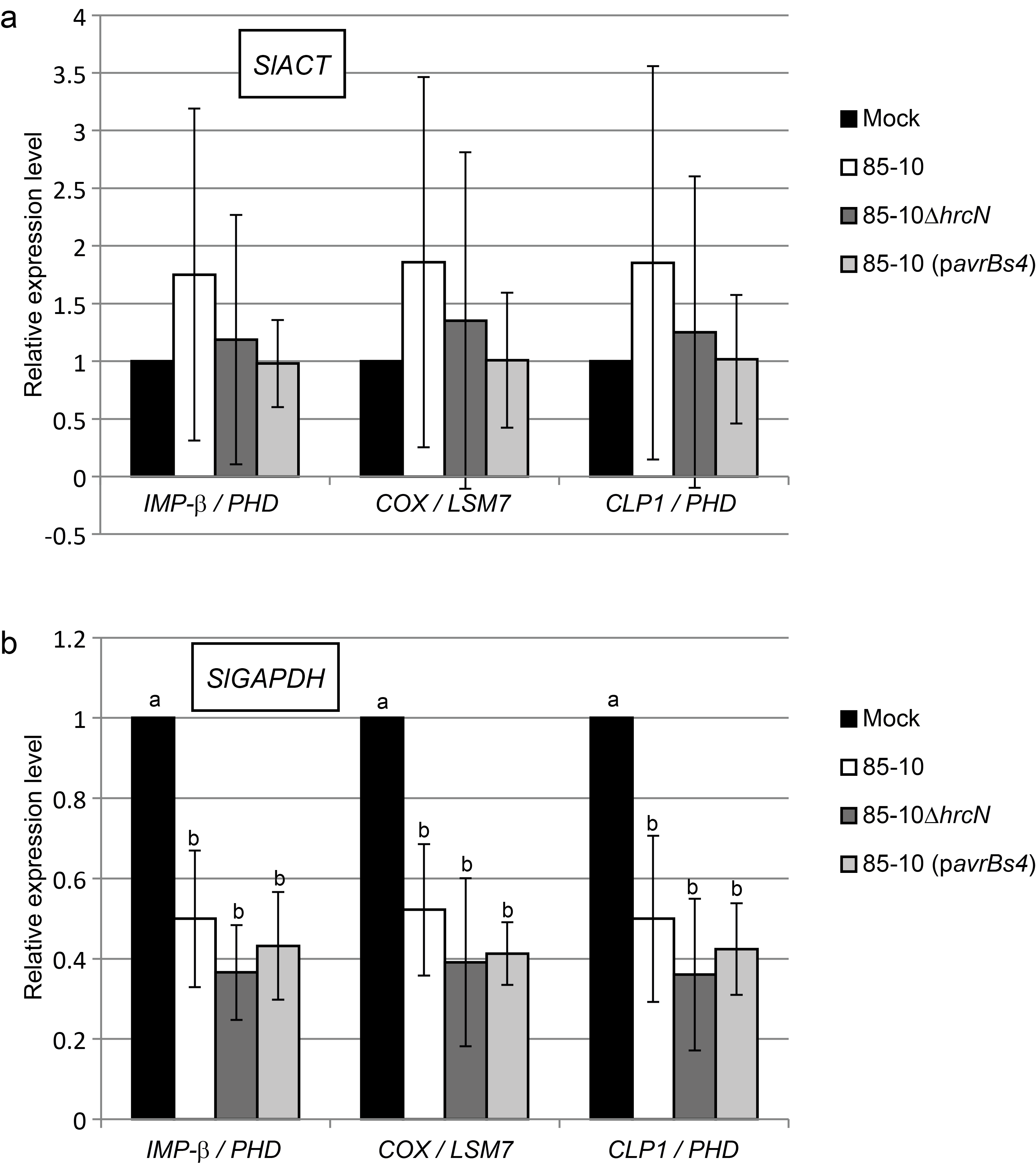

Supplement: S4 Fig — Expression patterns of (a) SlACT and (b) SlGAPDH in S. lycopersicum cv. MM leaves 6 hpi of 10 mM MgCl2 (mock) or 5×10^8^ cfu/ml of Xcv 85–10, 85–10ΔhrcN and 85-10(pavrBs4), respectively. qRT-PCR data were normalized with different reference gene pairs. Values are mean-fold changes in mRNA levels in Xcv-infected relative to mock-inoculated leaves for three biological replicates. Error bars indicate SD. Letters denote statistically significant differences (Student´s t-test, P < 0.05). (TIF) [file pone.0136499.s004.tif]

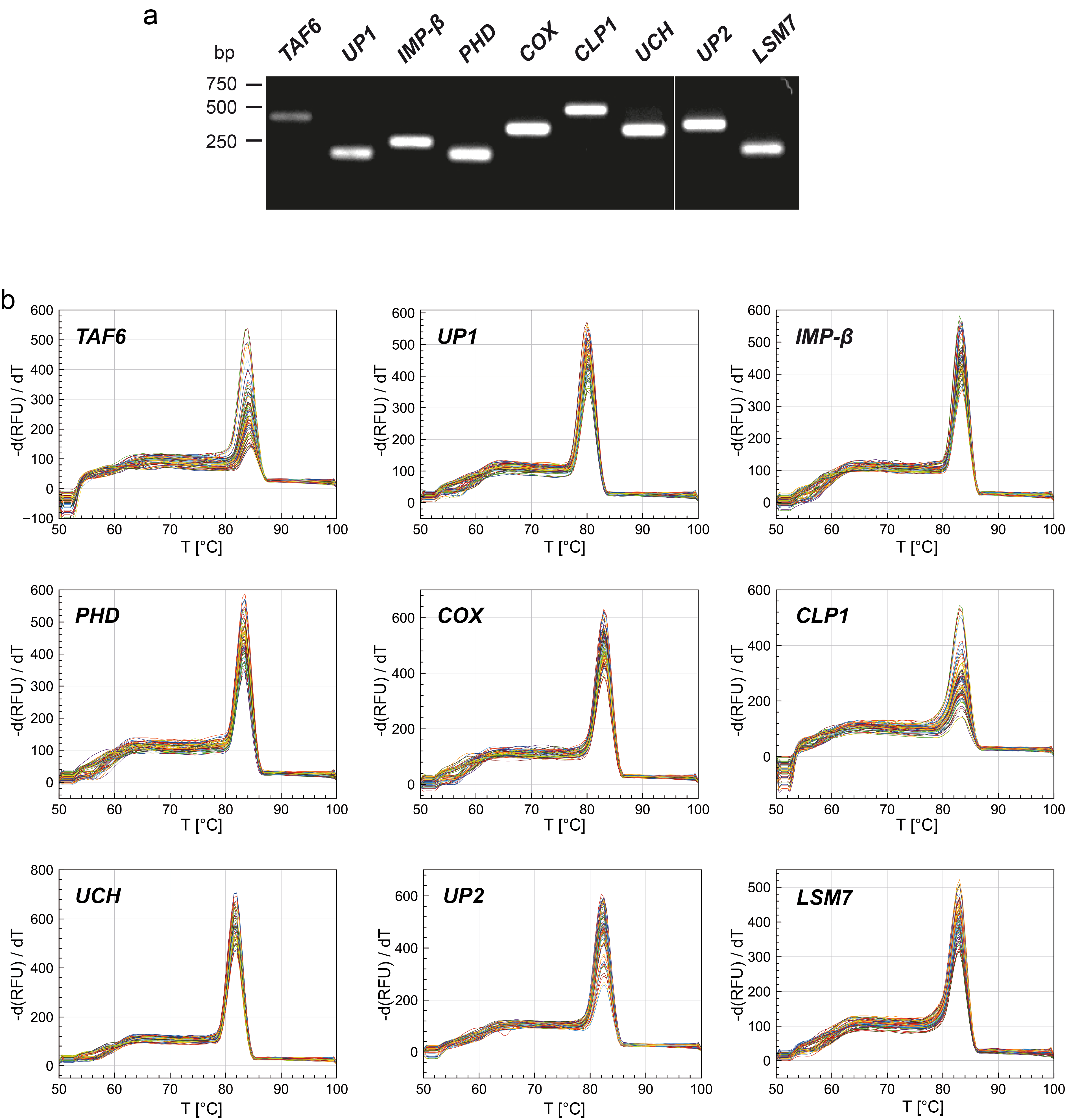

Supplement: S5 Fig — Presence of unique amplicons as a measure of PCR amplification specificity was determined (a) by electrophoresis on a 1% agarose gel and (b) by melting curve analysis. (TIF) [file pone.0136499.s005.tif]
