# Supplementary material for: Genome-Wide Identification and Validation of Reference Genes in Infected Tomato Leaves for Quantitative RT-PCR Analyses
Source: PLoS One. 2015 Aug 27;10(8):e0136499. doi: 10.1371/journal.pone.0136499 (PMC4552032; doi:10.1371/journal.pone.0136499)
Supplement: S1 Table — (DOC) [file pone.0136499.s006.doc]

**Table S1:** Oligonucleotide sequences used for qRT-PCR analyses.

| **Name** | **Accession number** | **Forward and reverse oligonucleotides**^a)^  **(5´-3´)** | **Ampli-con (bp)** | **PCR effi-ciency (%)** |
| --- | --- | --- | --- | --- |
| ***S. lycopersicum*** | | | | |
| *LRR22* | Solyc08g066210.2 | AAGATTGGAGGTTGCCATTGGAGC^b)^ | 82 | 95,89 |
|  |  | ATCGCGATGAATGATCGGTGGAGT^b)^ |  |  |
| *UGT* | Solyc09g092500 | GCAAAAGGGTTGATAGAGATCCAAAGG | 245 | 93,16 |
|  |  | CAATGAGGAAATGCCACCACAGGTAC |  |  |
| *ACT* | TC194780a | GAGCGTGGTTACTCGTTCA^b)^ | 287 | 83.41 |
|  |  | CTAATATCCACGTCACATTTCAT^b)^ |  |  |
| *GAPDH* | TC198136a | CTGCTCTCTCAGTAGCCAACAC^c)^ | 156 | 90.03 |
|  |  | CTTCCTCCAATAGCAGAGGTTT^c)^ |  |  |
| *UBI* | TC193502a | GGACGGACGTACTCTAGCTGAT^c)^ | 134 | 99.83 |
|  |  | AGCTTTCGACCTCAAGGGTA^c)^ |  |  |
| *EF-1α* | SGN-U212845 | GGTGGTTTTGAAGCTGGTATCTCC^d)^ | 455 | 96.56 |
|  |  | CCAGTAGGGCCAAAGGTCACA^d)^ |  |  |
| *TAF6* | Solyc10g006100.2.1 | CCAACTAAAGCGCTGCCACAAC | 426 | 95.25 |
|  |  | TGGTCCTTGTGTGCTTACTGGC |  |  |
| *UP1* | Solyc07g062920.2.1 | ACATGGAAGCTGTTAATGCCGGT | 185 | 99.32 |
|  |  | GATGTAGACCTTGTAGTTTACCTGTACCAT |  |  |
| *IMP-β* | Solyc01g111780.2.1 | TTGTTTGCTGCTTGGACCTGCT | 272 | 91.72 |
|  |  | GTTGGCCACAGAGATGGTTTCCT |  |  |
| *PHD* | Solyc06g051420.2.1 | GGGATGGGATGGAGCGTAGAGA | 279 | 98.76 |
|  |  | CATCACTCTCCTCTTGCAGCCT |  |  |
| *COX* | Solyc12g057120.1.1 | GCCTGCCACATCTGAAGAAAGC | 325 | 80.48 |
|  |  | GCCATTCTCCCTCTGCTCATTC |  |  |
| *CLP1* | Solyc01g009290.2.1 | GTCTTGGGTCAGGAAAAACTTTGCAG | 310 | 97.88 |
|  |  | CCATGTTAAGGTTGCCACAATTAAGTTTTTG |  |  |
| *UCH* | Solyc09g018730.2.1 | GGTGTGCAGGTTGAGGAGTTGT | 358 | 99.71 |
|  |  | GCACAAATGGCTCAGGTCTTGC |  |  |
| *PTBL* | Solyc02g088110.2.1 | GCTGCAAAGGACGCTTTAGAGG | 252 | 97.46 |
|  |  | AACAGCACCAGTAGCGGCATAG |  |  |
| *UP2* | Solyc08g060860.2.1 | TCGCTTGCATGGGAAGTTGTCT | 407 | 98.72 |
|  |  | AGGAAAAGGCTTGTTGGTCCCC |  |  |
| *LSM7* | Solyc09g009640.2.1 | GGTGGAAGACAAGTGGTTGGAACAC | 220 | 96.37 |
|  |  | CGTCTGGCTGAACAAAAGGATTGG |  |  |
| *ACP* | Solyc04g015370.2.1 | TCGAAACCCTAGGCTCATCGGA | 272 | 95.44 |
|  |  | TCTTGTCGGCTTCATTGTCTGGA |  |  |
| ***C. annuum*** | | | | |
| *LRR22* | XM_004245095 | AAGATTGGAGATAGCCATTGGAGC | 82 | 87.5 |
|  |  | ATCGCGATGAATGATTGGTGGAGT |  |  |
| *TFT4* | SGN-U198762 | TTAAAACTGGGGCAGAGAGGAAAG^e)^ | 134 | 91.71 |
|  |  | CACTGAAAAGTTAAGGGCAAGTCC^e)^ |  |  |
| *ACT* | AY572427.1 | TGTTATGGTAGGGATGGGTC^f)^ | 228 | 92.94 |
|  |  | TTCTCTCTATTTGCCTTGGG^f)^ |  |  |
| *β-TUB* | EF495259.1 | GAGGGTGAGTGAGCAGTTC^f)^ | 167 | 91.99 |
|  |  | CTTCATCGTCATCTGCTGTC^f)^ |  |  |
| *EF-1α* | AF242732 | AGTCAACTACCACTGGTCAC^g)^ | 205 | 91.77 |
|  |  | GTGCAGTAGTACTTAGTGGTC^g)^ |  |  |
| *GAPDH* | GAPCP-2 | ATGATGATGTGAAAGCAGCG^f)^ | 276 | 90.01 |
|  |  | TTTCAACTGGTGGCTGCTAC^f)^ |  |  |
| *TAF6* | CA10g01290 | CCAACTAAAGCGCTGCCACAAC | 426 | 72.09 |
|  |  | TGGTCCATGTGTGTTTACTGGC |  |  |
| *UP1* | CA07g17500 | ACACGGGAGCTGTTAATACCAGT | 185 | 99.32 |
|  |  | GATGTAGACCTTGTAGTTTACCTGTACCAT |  |  |
| *IMP-β* | CA08g19500 | TTGTTTGTTCCTTGGACCTGCT | 272 | 83.07 |
|  |  | GTTAGCCACAGAAATGGTTTCCT |  |  |
| *PHD* | CA00g95420 | AAGATGAAGAGGAGGGCTTGGATG | 196 | 87.44 |
|  |  | CAAGATGGACACTTGTACTGCTTAATATG |  |  |
| *COX* | CA12g10550 | GCTTGCTGCGCCTAAAGAAAGC | 325 | 89.24 |
|  |  | GCCATTTTCCCTCTGCTCATTC |  |  |
| *CLP1* | CA01g13750 | GTCTTGGGTCAGGAAAAACTTTGTAG | 504 | 89.39 |
|  |  | CCATGTTAAGGTTCCCACAATTAGATATTTG |  |  |
| *UCH* | CA09g10790 | GGTGTGCAGGTTGAGGAGTTGT | 358 | 96.01 |
|  |  | GCACAAATGGCTCCGGTCTTGC |  |  |
| *UP2* | CA08g03550 | TGTCTTGGGTGCTGTGTAAATCCTC | 407 | 89.41 |
|  |  | AGGGAATGACTTGTTGGTCCCC |  |  |
| *LSM7* | CA09g00800 | GGTGGAAGACAAGTGGTAGGAACAC | 220 | 87.76 |
|  |  | CGTCTGGCTGGAGAAAAGGATTCG |  |  |

^a)^Oligonucleotide sequences designed in this study or according to ^b)^[1], ^c)^[2], ^d)^[3], ^e)^[4], ^f)^[5], ^g)^[6]. Details on PCR profiles are available upon request.

R**eferences**

1. Taylor KW, Kim JG, Su XB, Aakre CD, Roden JA. Tomato TFT1 is required for PAMP-triggered immunity and mutations that prevent T3S effector XopN from binding to TFT1 attenuate *Xanthomonas* virulence. PLoS Pathog. 2012;8(6):e1002768. doi: 10.1371/journal.ppat.1002768.

2. Løvdal T, Lillo C. Reference gene selection for quantitative real-time PCR normalization in tomato subjected to nitrogen, cold, and light stress. Anal Biochem. 2009;387(2):238-42. doi: 10.1016/j.ab.2009.01.024.

3. Cohn JR, Martin GB. *Pseudomonas syringae* pv. *tomato* type III effectors AvrPto and AvrPtoB promote ethylene-dependent cell death in tomato. Plant J. 2005;44(1):139-54. doi: 10.1111/j.1365-313X.2005.02516.x.

4. Teper D, Salomon D, Sunitha S, Kim J-G, Mudgett MB, Sessa G. *Xanthomonas euvesicatoria* type III effector XopQ interacts with tomato and pepper 14–3–3 isoforms to suppress effector-triggered immunity. Plant J. 2014;77(2):297-309. doi: 10.1111/tpj.12391.

5. Wan H, Yuan W, Ruan M, Ye Q, Wang R, Li Z, et al. Identification of reference genes for reverse transcription quantitative real-time PCR normalization in pepper (*Capsicum annuum* L.). Biochem Biophys Res Commun. 2011;416(1-2):24-30. doi: 10.1016/j.bbrc.2011.10.105.

6. Bin WS, Wei LK, Ping DW, Li Z, Wei G, Bing LJ, et al. Evaluation of appropriate reference genes for gene expression studies in pepper by quantitative real-time PCR. Mol Breeding. 2012;30(3):1393-400. doi: 10.1007/s11032-012-9726-7.
