# Supplementary material for: Genome-Wide Identification and Validation of Reference Genes in Infected Tomato Leaves for Quantitative RT-PCR Analyses
Source: PLoS One. 2015 Aug 27;10(8):e0136499. doi: 10.1371/journal.pone.0136499 (PMC4552032; doi:10.1371/journal.pone.0136499)
Supplement: S2 Table — (DOC) [file pone.0136499.s007.doc]

**Table S2:** Functional classification of Arabidopsis orthologs corresponding to the new tomato reference genes.

| **Gene** | **GeneID** | ***A. thaliana* ortholog** | **Amino acid ident. (%)** | **Functional classification** | **Reference** |
| --- | --- | --- | --- | --- | --- |
| *TAF6* | Solyc10g006100.2.1 | AT1G04950 | 59 | DNA transcription | [1] |
| *IMP-β* | Solyc01g111780.2.1 | AT2G16950 | 74 | nuclear import | [2] |
| *PHD* | Solyc06g051420.2.1 | AT5G05610 | 69 | chromatin modification | [3] |
| *COX* | Solyc12g057120.1.1 | AT1G22450 | 59 | oxidation-reduction process | [4] |
| *CLP1* | Solyc01g009290.2.1 | AT3G04680 | 69 | mRNA splicing | [5] |
| *UCH* | Solyc09g018730.2.1 | AT1G65650 | 79 | cullin deneddylation, protein deubiquitination | [6] |
| *PTBL* | Solyc02g088110.2.1 | AT3G01150 | 80 | mRNA splicing | [7] |
| *LSM7* | Solyc09g009640.2.1 | AT2G03870 | 87 | splicosomal complex | [8] |
| *ACP* | Solyc04g015370.2.1 | AT1G65290 | 76 | fatty acid biosynthesis | [9] |

R**eferences**

1. Lago C, Clerici E, Dreni L, Horlow C, Caporali E, Colombo L, et al. The Arabidopsis TFIID factor AtTAF6 controls pollen tube growth. Dev Biol. 2005;285(1):91-100. doi: 10.1016/j.ydbio.2005.06.006.

2. Ziemienowicz A, Haasen D, Staiger D, Merkle T. Arabidopsis transportin 1 is the nuclear import receptor for the circadian clock-regulated RNA-binding protein AtGRP7. Plant Mol Biol. 2003;53(1-2):201-12. doi: 10.1023/B:PLAN.0000009288.46713.1f.

3. Lee WY, Lee D, Chung WI, Kwon CS. Arabidopsis ING and Alfin1-like protein families localize to the nucleus and bind to H3K4me3/2 via plant homeodomain fingers. Plant J. 2009;58(3):511-24. doi: 10.1111/j.1365-313X.2009.03795.x.

4. Ohtsu K, Nakazono M, Tsutsumi N, Hirai A. Characterization and expression of the genes for cytochrome c oxidase subunit VIb (COX6b) from rice and *Arabidopsis thaliana*. Gene. 2001;264(2):233-9. doi: 10.1016/s0378-1119(01)00334-1.

5. Zhao H, Xing D, Li QQ. Unique features of plant cleavage and polyadenylation specificity factor revealed by proteomic studies. Plant Physiol. 2009;151(3):1546-56. doi: 10.1104/pp.109.142729.

6. Tian G, Lu Q, Kohalmi SE, Rothstein SJ, Cui Y. Evidence that the Arabidopsis ubiquitin C-terminal hydrolases 1 and 2 associate with the 26S proteasome and the TREX-2 complex. Plant Signal Behav. 2012;7(11):1415-9. doi: 10.4161/psb.21899.

7. Simpson CG, Lewandowska D, Liney M, Davidson D, Chapman S, Fuller J, et al. Arabidopsis PTB1 and PTB2 proteins negatively regulate splicing of a mini exon splicing reporter and affect alternative splicing of endogenous genes differentially. New Phytol. 2014;203(2):424-36. doi: 10.1111/nph.12821.

8. Perez-Santángelo S, Mancini E. Role for *LSM* genes in the regulation of circadian rhythms. Proc Natl Acad Sci U S A. 2014;111(42):15166-71. doi: 10.1073/pnas.1409791111.

9. Li-Beisson Y, Shorrosh B, Beisson F. Acyl-lipid metabolism. In: The Arabidopsis book. American Society of Plant Biologists. 2013. p. e0161. doi: 10.1199/tab.0161.
